# Epigenetic regulation of Neuregulin 1 promotes breast cancer progression associated to hyperglycemia

**DOI:** 10.1038/s41467-023-36179-8

**Published:** 2023-01-27

**Authors:** Changhu Lee, Min Kim, Chanho Park, Woobeen Jo, Jeong Kon Seo, Sahee Kim, Jiyoung Oh, Chu-Sook Kim, Han Suk Ryu, Kyung-Hun Lee, Jiyoung Park

**Affiliations:** 1 Department of Biological Sciences, College of Information and Biotechnology, Ulsan National Institute of Science and Technology, Ulsan, 44919 Republic of Korea; 2 UNIST Central Research Facility, Ulsan National Institute of Science and Technology, Ulsan, 44919 Republic of Korea; 3 Department of Pathology, Seoul National University Hospital, Seoul National University College of Medicine, Seoul, Republic of Korea; 4 Department of Internal Medicine, Seoul National University Hospital, Cancer Research Institute, Seoul National University, Seoul, Republic of Korea

**Keywords:** Breast cancer, Diabetes complications, Imprinting

## Abstract

Hyperglycemia is a risk factor for breast cancer-related morbidity and mortality. Hyperglycemia induces *Neuregulin 1* (*Nrg1*) overexpression in breast cancer, which subsequently promotes tumor progression. However, molecular mechanisms underlying hyperglycemia-induced *Nrg1* overexpression remain poorly understood. Here, we show that hyperglycemia causes active histone modifications at the *Nrg1* enhancer, forming enhanceosome complexes where recombination signal binding protein for immunoglobulin kappa J region (RBPJ), E1A binding protein p300 (P300), and SET domain containing 1 A (SETD1A) are recruited to upregulate Nrg1 expression. Deletions in RBPJ-binding sites causes hyperglycemia-controlled *Nrg1* levels to be downregulated, resulting in decreased tumor growth in vitro and in vivo. Mice with modest-temporary hyperglycemia, induced by low-dose short-exposure streptozotocin, display accelerated tumor growth and lapatinib resistance, whereas combining lapatinib with N-[N-(3,5-difluorophenacetyl)-l-alanyl]-S42 phenylglycine t-butyl ester (DAPT) ameliorates tumor growth under these modest hyperglycemic conditions by inhibiting NOTCH and EGFR superfamilies. NOTCH activity is correlated with *NRG1* levels, and high *NRG1* levels predicts poor outcomes, particularly in HER2-positive breast cancer patients. Our findings highlight the hyperglycemia-linked epigenetic modulation of *NRG1* as a potential therapeutic strategy for treating breast cancer patients with diabetes.

## Abstract (web summary)

Despite hyperglycemia has been associated to breast cancer, the underlying mechanisms are not completely understood. Here, the authors show that epigenetic regulation of Nrg1 gene during hyperglycemia promotes breast cancer development.

## Introduction

Breast cancer remains the second leading cause of cancer-related deaths in Western countries, despite advances in diagnosis, treatment, and management^1^. Diabetes is a growing health issue, with patient numbers more than tripling in the past two decades^2^. Hyperglycemia, a hallmark of diabetes, is associated with increased risk of breast cancer development and poor disease outcome^3^. There have been studies on the effect of hyperglycemia on malignant tumor progression^4^; however, the molecular link between hyperglycemia and breast cancer risk remains largely unknown.

Hyperglycemia causes aberrant gene expression by altering the epigenome, a process called hyperglycemic memory^3^. This leads to aggressive tumor progression that persists even after glycemic control is therapeutically achieved. Neuregulin 1 (*Nrg1*) is a key mediator linking hyperglycemic memory in breast cancer cells with malignant tumor progression^5^. NRG1 belongs to the epidermal growth factor (EGF) family, contains an EGF-like domain, and is an HER3 (ERBB3) ligand. Multiple NRG1 isoforms are generated via alternative splicing and action of different promoters^6^ and distributed across multiple tissues, where they play important roles in proliferation, differentiation, and in developmental and pathological processes in the nervous system, heart, and epithelial cells^6^. Furthermore, through the NRG1-HER3 signaling axis, NRG1 contributes to malignant tumor development in certain cancer types, including gastric, pancreatic, and breast cancer, and its overexpression is closely associated with poor prognosis^7,8^. As such, *NRG1*-encoded proteins are oncogenic as they are mitogens capable of promoting tumor growth. Conversely, *NRG1* is also considered a tumor-suppressor gene as it is inactivated in most human breast cancer cells by DNA hypermethylation of the *NRG1* promoter region but is expressed in normal human mammary epithelial cells^9,10^. We previously reported that Nrg1 isoform type 1 (referred to here as Nrg1) is expressed at low levels in breast cancer cells, and its overexpression is switched on under hyperglycemic conditions. Moreover, through formaldehyde-assisted isolation of regulatory elements (FAIRE) sequencing, we identified a distal *Nrg1* enhancer region, whose chromatin structure exhibited an open configuration under hyperglycemic conditions^5^. However, little is known about the factors involved in *Nrg1* enhancer activation, and how they regulate *Nrg1* overexpression in breast cancer cells in patients with diabetes.

In this work, we dissect the molecular mechanisms underlying epigenetic regulations of the *Nrg1* enhancer that drive *Nrg1* overexpression in breast cancer cells under hyperglycemic conditions and define therapeutic strategies for overcoming drug resistance and breast cancer recurrence in patients with diabetes. We used DNA-protein pull-down using *Nrg1* enhancer sequence as bait followed by LC/MS and identified RBPJ as a key component of the *Nrg1* enhanceosome. High glucose-adapted cancer cells and STZ-induced modest-temporary hyperglycemia mouse models were used for in vitro and in vivo studies, respectively. We also analyzed public databases and our own data from patients with breast cancer to validate results of the in vitro and in vivo studies.

## Results

### Hyperglycemia upregulates *Nrg1* and promotes cancer cell proliferation

**Fig. 1:**
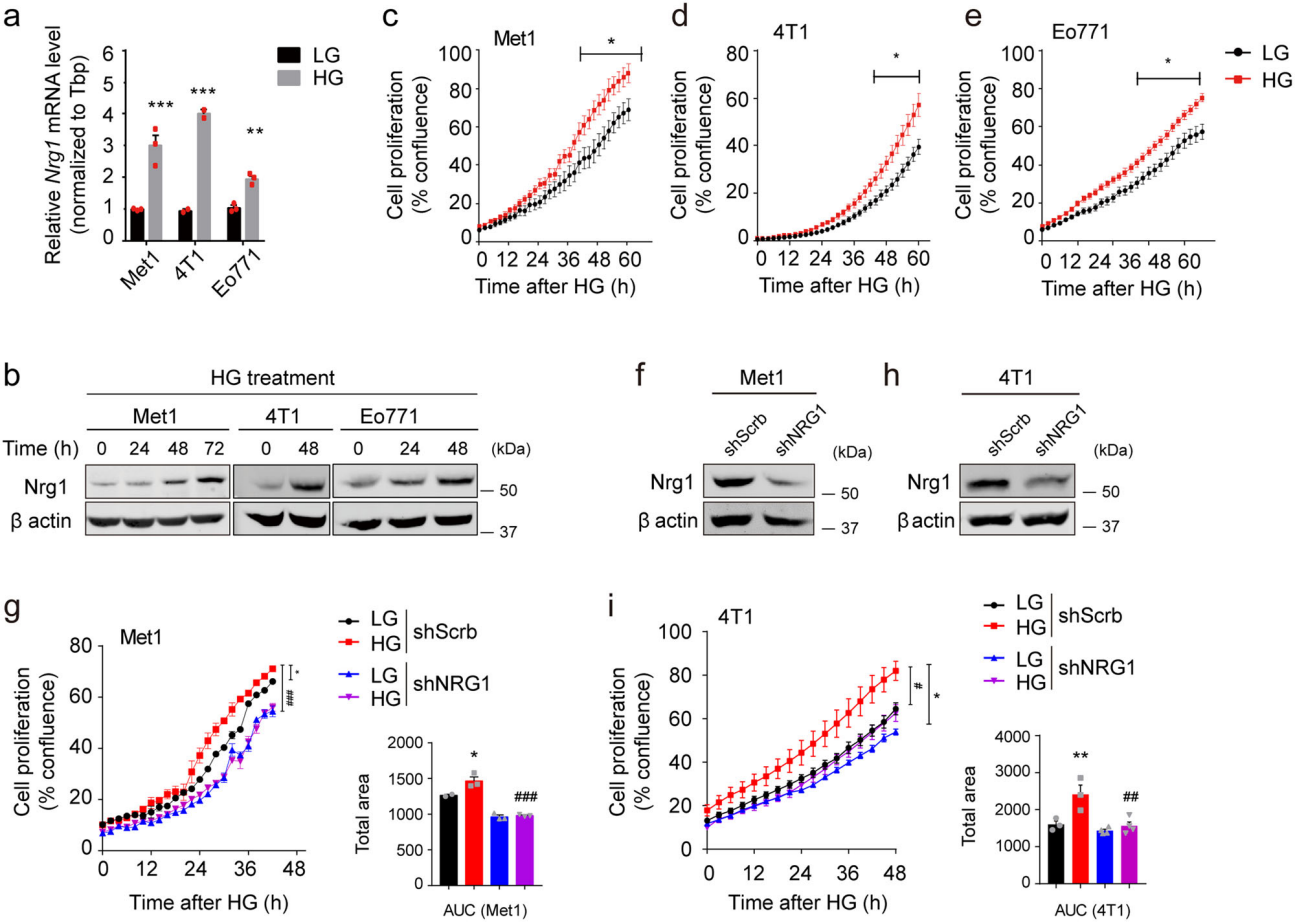
Hyperglycemia induces *Nrg1* expression and promotes cancer cell proliferation. **a** Relative mRNA levels of *Nrg1* in Met1, 4T1, and Eo771 breast cancer cells treated with either low glucose (LG) or high glucose (HG). **b** Time-course western blot analysis of Nrg1 and β actin protein levels in the breast cancer cell lines. **c–e** Analysis of proliferation of breast cancer cells treated with either LG or HG. **f, h** Western blot analysis of NRG1 in shScrb- or shNRG1-expressing Met1 or 4T1 cancer cells, and **g, i** analysis of their proliferation upon HG treatment and quantification of area under curve (AUC). Data represented mean ±SEM of technical replicates in **a**, and biological replicates in **c–e**, **g**, **i**, and *n* = 3 independent experiments were performed. Statistical significance was evaluated using two-way ANOVA followed by Bonferroni’s **a**, **c–e** and Tukey’s **g**, **i** post hoc test. ^*^P < 0.05; ^**^P < 0.01; ^***^P < 0.001 for column 1 versus other columns and ^#^*P* < 0.05; ^##^*P* < 0.01; ^###^*P* < 0.001 for column 2 versus column 4, **a** (from left to right, ^***^P < 0.0001, ^***^P < 0.0001, ^**^P = 0.0092), **c–e** (from **c** to **e**, ^*^P = 0.034, ^*^P = 0.0113, ^*^P = 0.0043) and **g** (^*^P = 0.03, ^###^*P* = 0.0001), **i** (^**^P = 0.0092, ^##^*P* = 0.0044). Source data are provided in Source Data file.

Most cancer cell lines are maintained in culture media containing high glucose (450 mg/dL, high glucose DMEM, HG), which have approximately 4‒5 times higher glucose concentration than in physiological circulation (80–110 mg/dL). Therefore, a low glucose (100 mg/mL, low glucose DMEM, LG) adaptation period is required to establish a cell line-based model system analyzing the cancer cell behaviors induced by hyperglycemia. For our in vitro experiments, mouse mammary tumor cell lines Met1, 4T1, and Eo771 were maintained in media containing LG for three days, and Nrg1 levels were monitored in 4T1 cells during the adaptation period. Nrg1 levels were decreased over three days of LG treatment (Supplementary Fig. 1a, b). After the three-day LG adaptation period, LG was replaced with HG for 7 days, and *Nrg1* mRNA levels in 4T1 cells increased (Supplementary Fig. 1c), suggesting that three days of LG adaptation is optimum for achieving basal Nrg1 levels required for response to HG treatment. Similar responses were observed in the other breast cancer cell lines. Thus, we validated that Nrg1 mRNA and protein levels in Met1, 4T1, and Eo771 cells are increased under HG (Fig. 1a, b). This increase was not associated with osmotic pressure, as Nrg1 levels were not altered by mannitol (Supplementary Fig. 1d, e), indicating that HG is an independent contributor to Nrg1 overexpression in breast cancer cells. As HG directly or indirectly affects several signaling pathways that promote cancer cell proliferation, including WNT/β-catenin, and hexosamine biosynthetic pathway (HBP)^4,11^, we confirmed that cancer cell proliferation is significantly elevated under HG versus LG conditions (Fig. 1c–e). However, these HG-driven cell viabilities were attenuated by *Nrg1* knockdown in Met1 and 4T1 cancer cells (Fig. 1f–i). These results suggest that HG supports cancer cell proliferation partially through an increased level of NRG1^12^.

### Hyperglycemia imprints active enhancer histone marks on *Nrg1* enhancer

**Fig. 2:**
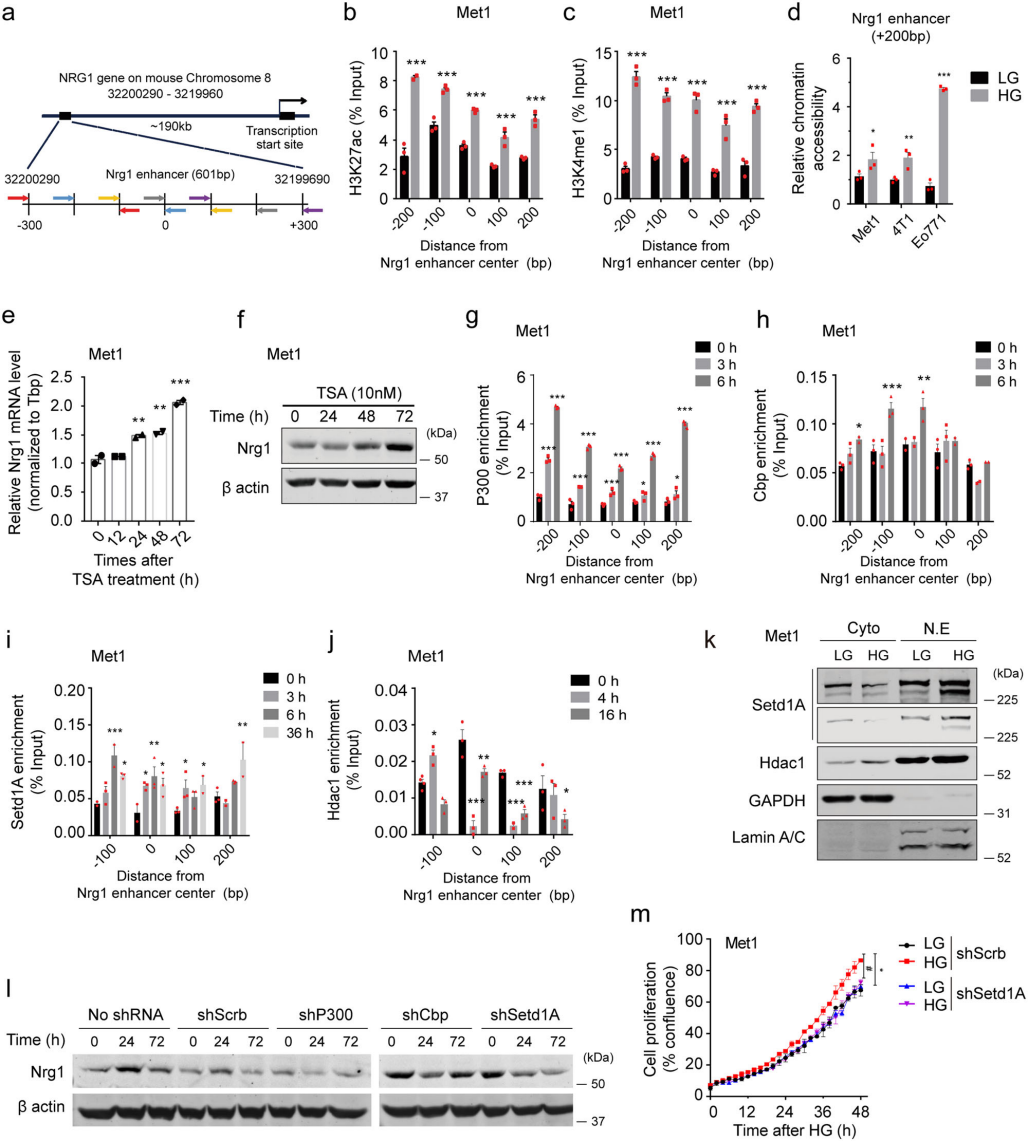
Hyperglycemia establishes active enhancer histone marks on the *Nrg1* enhancer. **a** Graphic representation of *Nrg1* gene and its enhancer. The *Nrg1* enhancer spans 0.6 kb and is located on mouse Chromosome 8: 32,200,290–32,199,690. The center of the enhancer is marked “0”, and primer pairs are shown in identical colors. **b, c** ChIP-qPCR analysis of H3K27ac and H3K4me1 enrichment at the *Nrg1* enhancer in Met1 cells treated with low glucose (LG) or high glucose (HG). **d** qPCR analysis of chromatin accessibility of the *Nrg1* enhancer (+200 bp) in Met1, 4T1, and Eo771 cancer cells. **e, f** mRNA and protein levels of Nrg1 in trichostatin A (TSA)-treated Met1 cells under LG. **g–j** ChIP-qPCR analysis of time-course binding occupancy of P300, CBP, SETD1A, and HDAC1 at the *Nrg1* enhancer in HG-treated Met1 cells. **k** Western blot analysis of subcellular protein localization of SETD1A and HDAC1 in LG- and HG-treated Met1 cells. **l** Protein levels of NRG1 in non-treated, shScrb, shP300, shCbp, and shSetd1A Met1 cells after up to 72 h of HG treatment. **m** Cellular proliferation of HG-treated cancer cells expressing scrambled or Setd1A shRNA. Data represented mean ±SEM of technical replicates in **b–e, g–j**, and biological replicates in **m**, and *n* = 3 independent experiments were performed. Statistical significance was evaluated using two-way ANOVA followed by Bonferroni’s **b, c**, **d**, Tukey’s **g**, **h**, **j**, **m**, and Dunnett’s **i** post hoc tests, and one-way ANOVA followed by Tukey’s post hoc test **e**. ^*^P < 0.05; ^**^P < 0.01; ^***^P < 0.001 for column 1 versus other columns and ^#^*P*  < 0.05; ^##^*P* < 0.01; ^###^*P* < 0.001 for column 2 versus column 4, **b**, **c** (all ^***^P < 0.0001), **d** (from left to right, ^*^P = 0.0413, ^**^P = 0.0088, ^***^P < 0.0001), **e** (from left to right, ^**^P = 0.0042, ^**^P = 0.0026, ^***^P < 0.0001), **g** (from left to right, ^***^P < 0.0001, ^*^P = 0.0367, ^*^P = 0.0189), **h** (from left to right, ^*^P = 0.0326, ^***^P = 0.0002, ^**^P = 0.0023), **i** (from left to right, ^***^P = 0.0004, ^*^P = 0.0226, ^*^P = 0.0276, ^**^P = 0.0025, ^*^P = 0.0239, ^*^P = 0.0407, ^*^P = 0.0358, ^**^P = 0.0025), **j** (from left to right, ^*^P = 0.0313, ^***^P < 0.0001, ^*^P = 0.0184, ^***^P = 0.0006, ^***^P = 0.0028, ^*^P = 0.0264), **m** (^*^P = 0.0141, ^#^*P* = 0.025). Source data are provided in Source Data file.

As *NRG1* expression is suppressed by DNA methylation of promoter regions in most breast cancers^9,13^, we used bisulfite sequencing to determine whether the DNA methylation status of CpG sites within the *Nrg1* enhancer region is modulated by hyperglycemic cues. The *Nrg1* enhancer spans 0.6 kb and is located approximately 190 kb from the *Nrg1* transcription start site; the position at the center of the *Nrg1* enhancer is denoted as “0” (Fig. 2a). A total of nine CpG sites were found in the *Nrg1* enhancer, most of which were unmethylated in Met1, 4T1, and Eo771 cancer cells under both LG and HG conditions, suggesting that DNA methylation is not associated with HG-induced upregulation of Nrg1 (Supplementary Fig. 2a). We also analyzed *Nrg1* enhancer histone modifications. Active enhancer marks, histone H3 lysine 4 mono-methylation (H3K4me1) and histone H3 lysine 27 acetylation (H3K27ac), were examined using ChIP-qPCR, revealing that HG increased H3K27ac and H3K4me1 enrichments within the enhancer (Fig. 2b, c). Consistent with active histone marks status, chromatin accessibility of the *Nrg1* enhancer (+200 bp) was also increased by HG treatment in breast cancer cells, suggesting that HG resulted in an open chromatin landscape within the *Nrg1* enhancer region. (Fig. 2d). Inhibiting histone deacetylase (HDAC) using trichostatin A (TSA) significantly increased Nrg1 mRNA and protein expression in LG-adapted Met1 and 4T1 cells (Fig. 2e, f and Supplementary Fig. 2b, c), suggesting that H3K27 histone acetylation may upregulate *Nrg1* expression. In agreement with previous reports that distal regulatory regions have low CpG methylation and are inversely correlated with active enhancer histone signatures^14^, DNA methylation was rarely observed along the *Nrg1* enhancer, whereas active enhancer histone marks were enriched in the enhancer region upon HG exposure.

To identify specific chromatin remodeling enzymes involved in *Nrg1* overexpression under hyperglycemic conditions, we investigated the recruitment of chromatin remodelers, such as HATs, HDAC, and HMT, to the *Nrg1* enhancer using ChIP-qPCR following HG treatment (Fig. 2g–j). P300 enhancer occupancy was increased for six hours, while CBP occupancy was increased to a lesser extent following HG treatment (Fig. 2g, h). HG also increased SETD1A binding occupancy along the *Nrg1* enhancer (Fig. 2i). Meanwhile, HDAC1 was displaced from the *Nrg1* enhancer 4–16 h after HG treatment (Fig. 2j). Nuclear SETD1A levels were elevated, while cytoplasmic HDAC1 levels were unaffected by HG, suggesting that HG affects nuclear SETD1A protein levels and potentially contributes to histone methylation within the active *Nrg1* enhancer (Fig. 2k). We confirmed the involvement of P300/CBP and SETD1A in HG-induced *Nrg1* upregulation using shRNA, and target gene knockdown efficiency was confirmed by western blot analysis (Supplementary Fig. 2d–f). HG-induced *Nrg1* overexpression was abolished by silencing of *P300, Cbp*, and *Setd1A* genes in Met1 cancer cells compared with scrambled (Scrb) shRNA or shRNA non-treated control cells (Fig. 2l), indicating that P300, CBP, and SETD1A may be associated with Nrg1 enhanceosome assembly. Additionally, HG-induced cellular proliferation was reduced by SETD1A depletion in Met1 cancer cells (Fig. 2m), confirming that SETD1A plays a pro-tumorigenic role in breast cancer^15,16^. These results indicated that multiple chromatin remodelers, including HDAC1, P300/CBP, and SETD1A, are involved in HG-linked chromatin remodeling. HDAC1 was dissociated and P300/CBP and SETD1A were recruited to the *Nrg1* enhanceosome complex to upregulate *Nrg1* expression following HG treatment.

### *Nrg1* expression is upregulated by NOTCH signaling via RBPJ recruitment to their active enhancer under hyperglycemic conditions

Signal-dependent transcription factors collaboratively fine-tune enhancer activities by dynamically orchestrating an enhanceosome assembly on chromatin in response to various environmental stimuli in association with histone modifiers^17^. Based on this, we used a DNA pull-down assay to analyze the specific transcriptional (co)factors that bind to the *Nrg1* active enhancer in response to hyperglycemia. Briefly, nuclear extracts of Met1 cancer cells grown under LG or HG conditions were incubated with a synthetic *Nrg1* enhancer oligonucleotide tagged with biotin as bait, and binding proteins were eluted using streptavidin beads and identified using mass spectrometry (Supplementary Fig. 3a). Thousands of candidates were enriched in the *Nrg1* enhancer sequence following HG treatment (Supplementary Data 1), and five candidates were selected following a literature search (Supplementary Fig. 3b). Recombination signal-binding protein for immunoglobulin kappa J region (*Rbpj*) is a key downstream effector of canonical Notch signaling pathway that interacts with GATA binding protein 4 (*Gata4*) and C-terminal binding protein 1 (*CtBP1*) following Notch activation^18,19^. Thus, their ability to regulate *Nrg1* expression was validated by siRNA-mediated knockdown (Supplementary Fig. 3c). Strikingly, Nrg1 mRNA and protein levels were decreased in siRNA-Rbpj transfected Met1 and Eo771 cells compared with that in controls (Supplementary Fig. 3d–f). We selected *Rbpj* for further analysis as a potential factor involved in Nrg1 enhanceosome assembly.

**Fig. 3:**
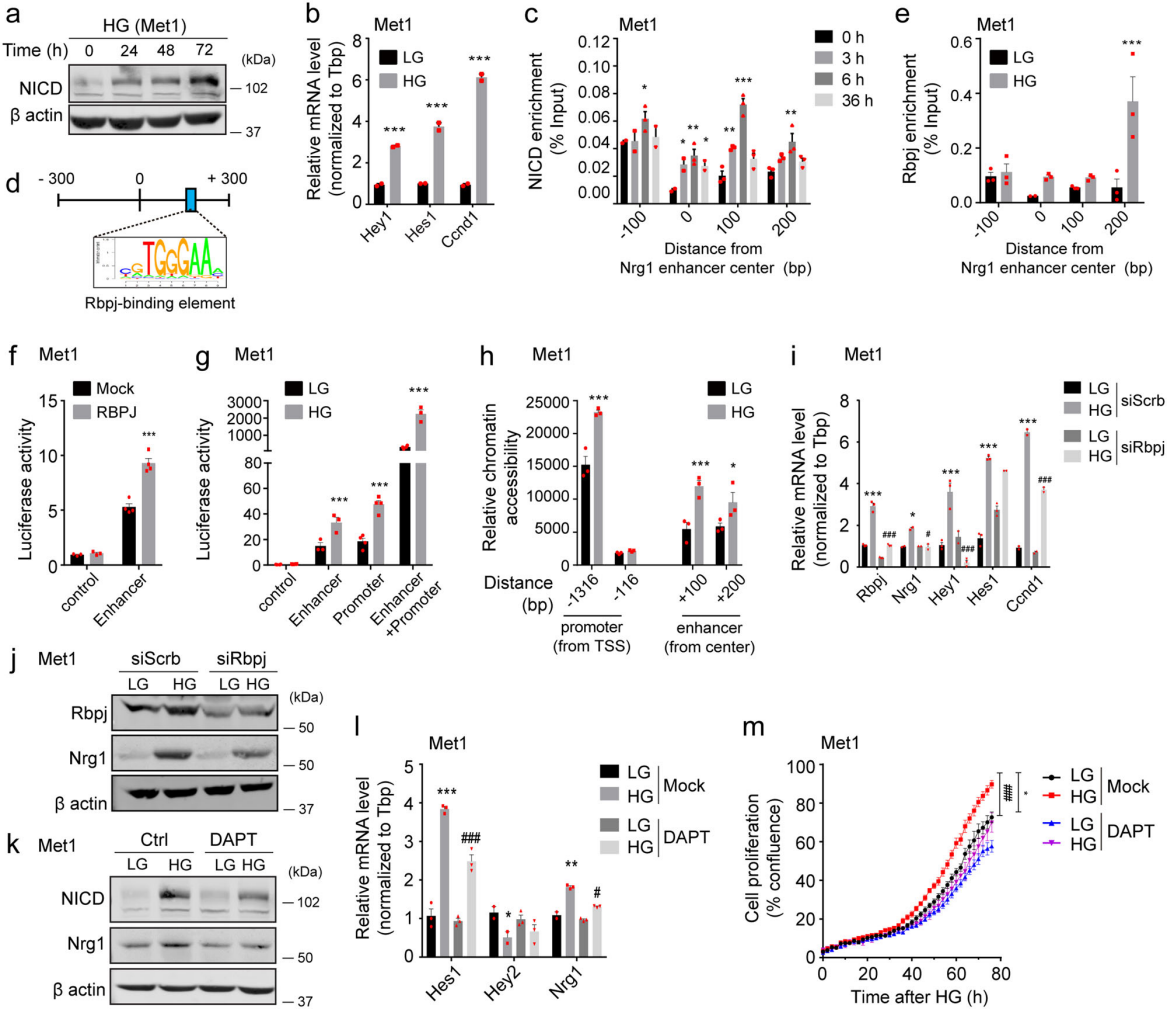
Hyperglycemia promotes Notch activation and Rbpj binding to the *Nrg1* enhancer. **a** Time-course western blot analysis of NOTCH intracellular domain (NICD) protein levels and **b** mRNA levels of Notch target genes in high glucose (HG)-treated Met1 cells. **c** ChIP-qPCR analysis of NICD binding occupancy at the *Nrg1* enhancer in Met1 cells. **d** Graphic representation of the RBPJ-binding site on the *Nrg1* enhancer (+108/114 bp). **e** ChIP-qPCR analysis of RBPJ binding occupancy on the *Nrg1* enhancer in LG- or HG-treated Met1 cells. **f** Luciferase reporter activity of the pGL4.23 vectors containing either control or the *Nrg1* enhancer in Met1 cells transfected with RBPJ. **g** Luciferase reporter activity of the pGL4.23 reporter vectors containing control, enhancer, 1 kb promoter, or enhancer with 1 kb promoter in Met1 cells treated with LG or HG. **h** Chromatin accessibility of the *Nrg1* promoter (−1316, −116 bp from TSS) and enhancer region (+100, +200 bp from center of the enhancer) in the Met1 cells treated with LG or HG. **i** mRNA levels of *Rbpj* and Notch target genes, and **j** NRG1 and RBPJ protein levels in LG- or HG-treated Met1 cells transfected with scrambled or Rbpj-targeting siRNA. **k** Expression of NRG1 and NICD proteins, **l** mRNA levels of Notch target genes, and **m** cellular proliferation in LG- or HG-treated Met1 cells following vehicle or DAPT treatment. Data represented mean ± SEM of technical replicates in **b-c, e–i, l**, and biological replicates in **m**, and *n* = 3 independent experiments were performed. Statistical significance was evaluated using two-way ANOVA followed by Bonferroni’s **b**, **e**, **f**, **g**, **h**, Dunnett’s **c**, and Tukey’s **i**, **l**, **m** post hoc tests. ^*^P < 0.05; ^**^P < 0.01; ^***^P < 0.001 for column 1 versus other columns and ^#^*P* < 0.05; ^##^*P* < 0.01; ^###^*P* < 0.001 for column 2 versus column 4, **b** (all ^***^P < 0.0001), **c** (from left to right, ^*^P = 0.0292, ^*^P = 0.0269, ^**^P = 0.001, ^*^P = 0.0376, ^**^P = 0.0026, ^***^P < 0.0001, ^**^P = 0.0014), **e** (^***^P < 0.0001), **f** (^***^P < 0.0001), **g** (from left to right, ^***^P = 0.0005, ^***^P < 0.0001, ^***^P < 0.0001), **h** (from left to right, ^***^P < 0.0001, ^***^P = 0.0004, ^*^P = 0.0434), **i** (from left to right, ^***^P < 0.0001, ^###^*P* < 0.0001, ^*^P = 0.0124, ^#^*P* = 0.0108, ^***^P < 0.0001, ^###^*P* < 0.0001, ^***^P < 0.0001, ^***^P < 0.0001, ^###^*P* < 0.0001), **l** (from left to right, ^***^P < 0.0001, ^###^*P* < 0.0001, ^**^P = 0.0091, ^**^P = 0.0014, ^#^*P* = 0.0116), **m** (^*^P = 0.0191, ^##^*P* = 0.0006). Source data are provided in Source Data file.

Notch signaling is initiated by NOTCH binding to its ligand. Upon activation, the NOTCH intracellular domain (NICD) is cleaved by γ-secretase, released from the cellular membrane, and translocates into the nucleus where it binds to RBPJ^20^. The NICD-RBPJ complex transcriptionally activates Notch target genes, such as the Hes family bHLH transcription factor (HES) or hairy/enhancer-of-split related with YRPW motif (HEY). NICD displaces corepressors while chromatin regulators and coactivators are recruited to the NICD-RBPJ complex, inducing chromatin remodeling^20^. Notch signaling pathway dysregulation is associated with multiple human diseases, including congenital and neurodegenerative disorders, diabetes, and certain cancers^21^. We examined whether the Notch signaling pathway is involved in Nrg1 upregulation in breast cancer cells under hyperglycemic conditions. NICD levels and Notch target genes, including *Hey1, Hes1*, and cyclin D1 (*Ccnd1*), were significantly upregulated in HG-treated Met1 cancer cells (Fig. 3a, b), suggesting that HG activates Notch signaling. Similar findings were observed in 4T1 cells (Supplementary Fig. 4a, b). NICD was recruited to the *Nrg1* enhancer region in HG-treated Met1 cancer cells (Fig. 3c). As putative RBPJ-binding sequences (TGGGAA) were found within the *Nrg1* enhancer (Fig. 3d), we analyzed whether RBPJ is recruited to the *Nrg1* enhancer by HG. HG increased RBPJ binding occupancy around the +200 bp position of *Nrg1* enhancer where the putative RBPJ binding site is located (Fig. 3e). Moreover, a luciferase-based reporter assay revealed that *Nrg1* enhancer activity was increased by introduction of RBPJ, whereas the control reporter had no effect in the Met1 and 4T1 cells (Fig. 3f and Supplementary Fig. 4c). Additionally, we investigated whether the *Nrg1* promoter region is also involved in HG-driven *Nrg1* overexpression. Upon HG treatment, activity in the *Nrg1* enhancer and the 1 kb region of the *Nrg1* promoter both increased. Notably, a reporter vector containing both the promoter and enhancer element displayed increased activity in a synergistic manner (Fig. 3g and Supplementary Fig. 4d). In the same line, chromatin accessibility of the *Nrg1* promoter (−1316 bp) and enhancer (+100 and +200 bp) was also elevated by HG treatment in Met1 and 4T1 cells (Fig. 3h and Supplementary Fig. 4e). These results suggest that both the promoter and the enhancer work coordinately to regulate *Nrg1* expression in response to HG.

To analyze the role of Notch pathways in HG-induced *Nrg1* upregulation, Nrg1 levels were assessed following genetic and pharmacological inhibition of Notch pathways in breast cancer cells using siRNA-Rbpj and DAPT (N-[N-(3,5-Difluoroph enacetyl)-L-alanyl]-S-phenylglycine t-butyl ester), respectively. HG augmented the levels of *Rbpj*, *Nrg1*, and Notch target genes, including *Hey1*, *Hes1*, and *Ccnd1* in control cells. However, these increases were suppressed by transfection of siRNA-Rbpj (Fig. 3i). Similar results were observed when RBPJ and NRG1 protein levels were analyzed (Fig. 3j). Similarly, DAPT treatment reduced NICD protein levels, and HG-induced NRG1 overexpression was abolished by DAPT in Met1 and 4T1 cells (Fig. 3k and Supplementary Fig. 4f). At the mRNA level, the expression of *Nrg1* and Notch target genes decreased following DAPT treatment in HG-treated cancer cells (Fig. 3l and Supplementary Fig. 4g). To explore the effect of HG-induced Notch activity on tumor progression, cancer cell proliferation was measured in LG and HG-adapted cancer cells in the absence or presence of DAPT. HG-mediated increase in cell proliferation was reduced by DAPT treatment of breast cancer cells (Fig. 3m and Supplementary Fig. 4h–i). Our data suggest that Notch signals play a crucial role in hyperglycemia-induced Nrg1 overexpression in various breast cancer cells, resulting in malignant tumor growth.

### Hyperglycemia activates Notch signaling pathways by increasing Notch ligand levels and NOTCH1 *O*-GlcNAcylation

We postulated that hyperglycemia partly affects the binding efficiency of the Notch receptor and its ligand through O-GlcNAc modification of the NOTCH1 receptor, which strengthens the interactions. To determine O-GlcNAc levels of NOTCH1 following HG treatment, immunofluorescence staining was performed without nuclear permeabilization to exclude NICD staining. HG increased O-GlcNAcylated NOTCH1 levels in Met1 cells (Supplementary Fig. 5a, b). To analyze the direct effects of O-GlcNAcylation on Notch activation and Nrg1 levels in breast cancer cells, we used O-(2-Acetamido-2-deoxy-D-glucopyranosylidene) amino-Z-N-phenylcarbamate (PUGNAc), an inhibitor of O-GlcNAcase (OGA) that causes a global increase in O-GlcNAcylation of substrate proteins. Global O-GlcNAcylated protein levels and Notch activity were elevated in Met1 cells, based on NICD and NRG1 expression (Supplementary Fig. 5c). PUGNAc treatment significantly elevated mRNA levels of Notch target genes and *Nrg1* compared to vehicle-treated cells (Supplementary Fig. 5d), suggesting that O-GlcNAcylation of NOTCH1 potentially switched on Notch activation, mimicking HG conditions. We also investigated whether Notch ligands are affected by hyperglycemia. Met1 cells were incubated with LG or HG, and Notch ligand mRNA levels were analyzed by qPCR. The levels of delta-like canonical Notch ligand 3 (*Dll3*) and delta like canonical Notch ligand 4 (*Dll4*) were elevated in response to HG (Supplementary Fig. 5e). These results strongly indicate that hyperglycemia augments Notch signaling pathways through increase in O-GlcNAcylation of NOTCH1 and an increase in Notch ligands such as *Dll3* and *Dll4*. Thus, hyperglycemia-induced *Nrg1* overexpression in breast cancer cells may be partly mediated by Notch activation, leading to NICD-RBPJ assembly in the active *Nrg1* enhanceosome.

### Nrg1 overexpression is caused by hyperglycemia-induced NICD-RBPJ recruitment to the *Nrg1* enhanceosome

**Fig. 4:**
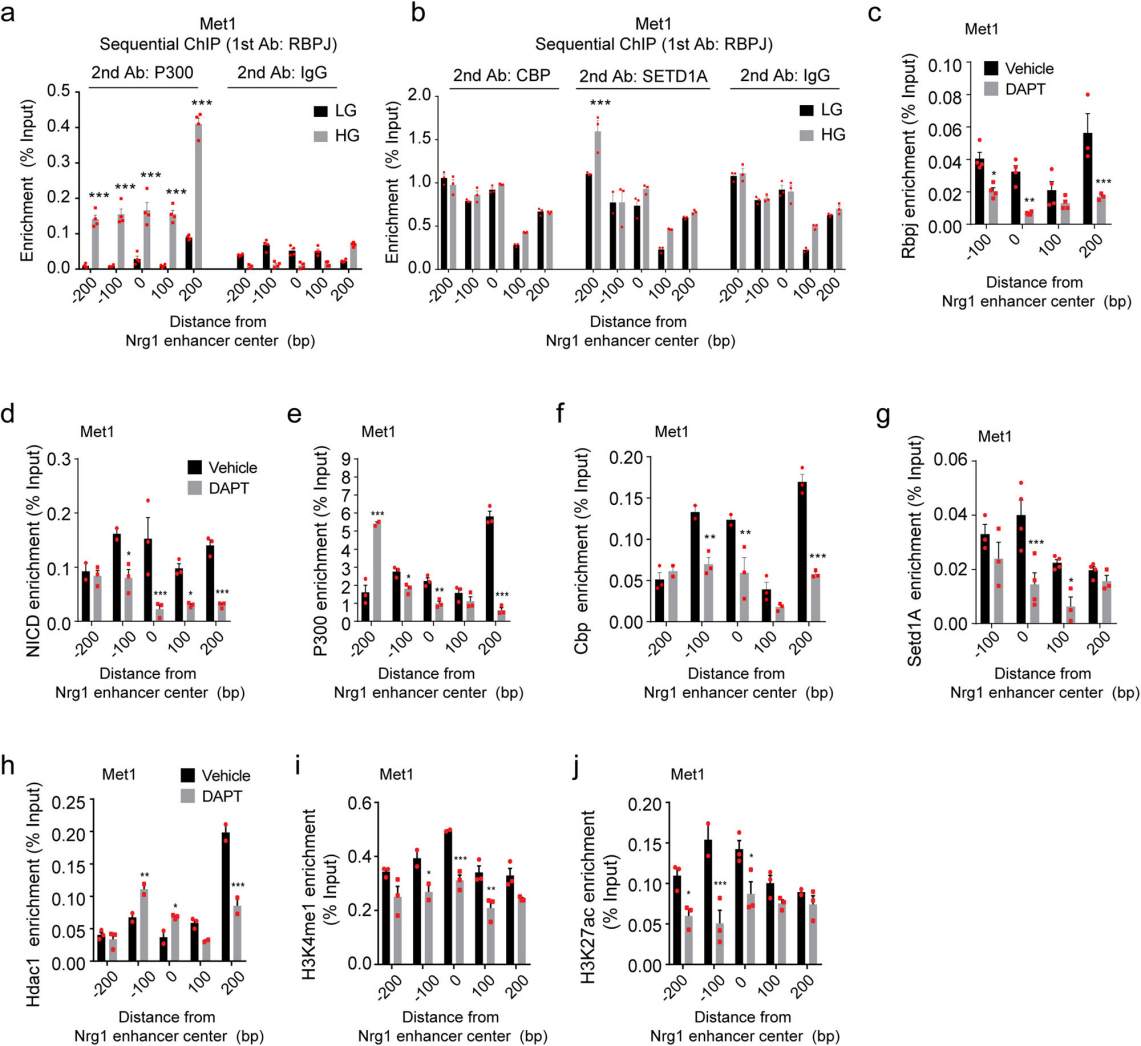
Sustained Notch activation is required to maintain the enhanceosome. **a, b** Sequential ChIP-qPCR analysis of binding co-occupancy of RBPJ with P300, CBP, and SETD1A within the *Nrg1* enhancer region in Met1 cancer cells treated with LG or HG. **c–j** ChIP-qPCR analysis of binding occupancy of RBPJ, NOTCH intracellular domain (NICD), P300, CBP, SETD1A, HDAC and enrichment of H3K4me1 and H3K27ac within the *Nrg1* enhancer region in Met1 cancer cells treated with vehicle or DAPT under HG. Data represented mean ± SEM of technical replicates in **a–j**, and *n* = 3 independent experiments were performed. Statistical significance was evaluated using two-way ANOVA followed by Bonferroni’s post hoc test **a**–**j**. ^*^P < 0.05; ^**^P < 0.01; ^***^P < 0.001, **a** (all ^***^P < 0.0001), **b** (^***^P < 0.0001), **c** (from left to right, ^*^P = 0.0133, ^**^P = 0.0013, ^***^P < 0.0001), **d** (from left to right, ^*^P = 0.0234,^***^P < 0.0001, ^*^P = 0.0378, ^***^P = 0.0006), **e** (from left to right, ^***^P < 0.0001, ^*^P = 0.0403, ^**^P = 0.0049, ^***^P < 0.0001), **f** (from left to right, ^**^P = 0.0019, ^**^P = 0.0016, ^***^P < 0.0001), **g** (from left to right, ^***^P = 0.0003, ^*^P = 0.0323), **h** (from left to right, ^**^P = 0.0066, ^*^P = 0.0349, ^***^P < 0.0001), **i** (from left to right, ^*^P = 0.033, ^***^P = 0.0005, ^**^P = 0.0047), **j** (from left to right, ^*^P = 0.0279, ^***^P < 0.0001, ^*^P = 0.0126). Source data are provided in Source Data file.

Having shown that *Nrg1* overexpression is mediated by the Notch‒RBPJ axis under HG conditions, we investigated whether RBPJ interacted with other co-activators, such as P300, CBP, or SETD1A, and whether the Notch pathway is involved in the assembly of the *Nrg1* enhanceosome complex. To assess the interaction of RBPJ with P300, sequential ChIP assay was performed using anti-RBPJ antibody, followed by anti-P300 antibody. In Met1 cells, 6 h of HG treatment resulted in elevated binding co-occupancy of RBPJ with P300, suggesting that the interaction between RBPJ and P300 is facilitated by HG treatment **(**Fig. 4a**)**. Likewise, binding co-occupancy of RBPJ and SETD1A was also elevated at the −200 bp position of the Nrg1 enhancer after 48 h of HG treatment, whereas co-occupancy of RBPJ with CBP remained unchanged (Fig. 4b). Subsequently, alteration of DNA accessibility of various chromatin remodelers and active histone marks on the *Nrg1* enhancer was determined by ChIP-qPCR with or without Notch inhibition. HG-treated Met1 cells were treated with DAPT, and NICD-RBPJ binding occupancy with various chromatin remodelers, including P300, SETD1A, and HDAC1, at the *Nrg1* enhancer were analyzed. HG-induced recruitment of NICD-RBPJ, P300, CBP, and SETD1A at the *Nrg1* enhancer was diminished, whereas HG-induced HDAC1 dissociation was suppressed by DAPT (Fig. 4c–h). Consistent with the results observed using DAPT, the binding occupancy of NICD-RBPJ, P300, CBP, and SETD1A on the *Nrg1* enhancer regions decreased, while HDAC1 dissociation from the *Nrg1* enhancer was suppressed in siRNA-Rbpj transfected cells (Supplementary Fig. 6a–f). Enrichment of active histone marks, such as H3K4me1 and H3K27ac, at *Nrg1* enhancer regions was also determined following Notch inhibition. Consistent with accessibility of HAT and HMT, enrichment of active histone marks such as H3K4me1 and H3K27ac were decreased by either DAPT or siRNA-Rbpj (Fig. 4i, j and Supplementary Fig. 6g, h). siRNA-mediated Rbpj knockdown efficiency was confirmed by western blotting (Supplementary Fig. 6i).

To assess gain-of-function of Notch signals, NICD was overexpressed in 4T1 cells under LG, and Nrg1 levels were measured. Forced NICD overexpression in LG-treated 4T1 cells increased both protein and mRNA levels of Nrg1 in cancer cells compared to that in control cells (Supplementary Fig. 6j, k). Increased expression of Notch target genes due to NICD overexpression was confirmed using qRT-PCR (Supplementary Fig. 6k). ChIP-qPCR revealed that NICD overexpression promoted RBPJ binding occupancy across the *Nrg1* enhancer. Similarly, the two active enhancer histone modifications were enriched upon Notch activation (Supplementary Fig. 6l–n), suggesting that NICD overexpression is sufficient to establish active histone marks in the *Nrg1* enhancer and drive Nrg1 overexpression. These results point to a crucial role of the Notch-Rbpj axis in modulating the *Nrg1* enhanceosome complex, and the binding of chromatin remodelers to the *Nrg1* enhancer could be modulated by Notch signaling under HG conditions.

### *Nrg1* enhancer is required for hyperglycemia-induced malignant tumor progression

**Fig. 5:**
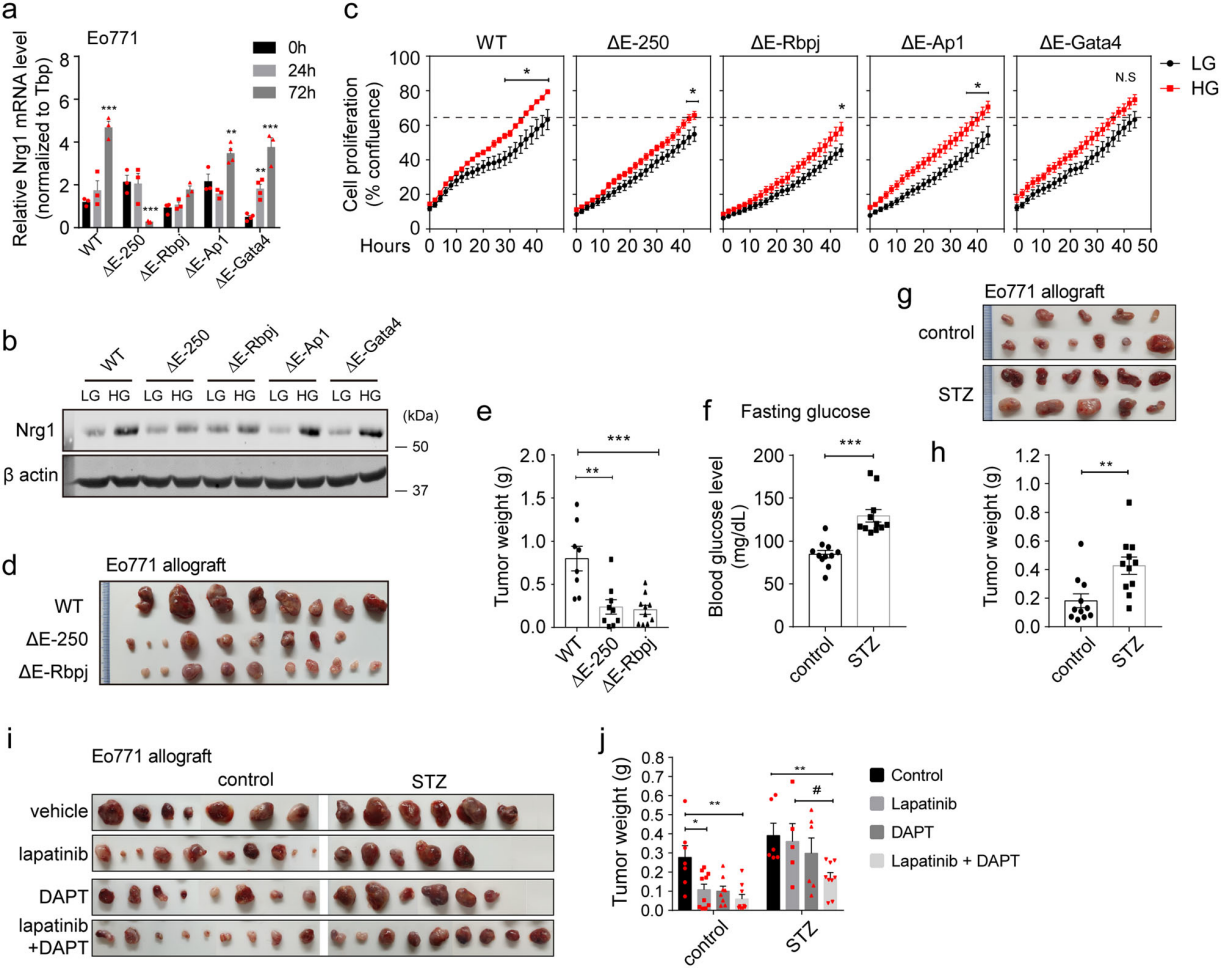
*Nrg1* enhancer is required for hyperglycemia-induced tumor progression, and dual inhibition of Notch and HER2 improves response to lapatinib under hyperglycemic conditions. **a** mRNA and **b** protein levels of Nrg1 and **c** cellular proliferation in low glucose (LG)- or high glucose (HG)-treated Eo771 cells lacking parts of the *Nrg1* enhancer elements, as indicated. **d, e** Representative images of tumors and their weights (*n* = 8, 9, 10 in each group, respectively). Tumors were generated from the indicated Eo771 genotypes. **f–h** Eo771 cancer cells were subcutaneously implanted into either normal or STZ-induced modest-temporary hyperglycemic mice. **f** Fasting blood glucose levels in mice. **g, h** Representative images of tumors and their weights from vehicle or STZ-treated mice (*n* = 11 in each group). **i, j** Lapatinib and DAPT combination treatments were administered to normal STZ-induced modest-temporary hyperglycemic mice (*n* = 7, 10, 8, 9 for control and 6, 5, 6, 6 for STZ group, respectively). **i** Representative images and weights **j** of tumors in the indicated groups. Data represented mean ± SEM of technical replicates in **a**, and biological replicates in **c, e, f, h, j**, and *n* = 3 independent experiments were performed in **a–h**, *n* = 1 for **i**, **j**. Statistical significance was evaluated using two-way ANOVA followed by Tukey’s **a**, **j** and Bonferroni’s **c** post hoc test, and one-way ANOVA followed by Tukey’s post hoc test **e**, and two-tailed Student’s *t*-test **f**, **h**. ^*^P < 0.05; ^**^P < 0.01; ^***^P < 0.001 for column 1 versus others, and ^#^*P* < 0.05; ^##^*P* < 0.01; ^###^*P* < 0.001 for column 2 versus column 4, **a** (from left to right, ^***^P < 0.0001, ^***^P < 0.0001, ^**^P = 0.0024, ^***^P = 0.001, ^***^P < 0.0001), **c** (from left to right, ^*^P = 0.0277, ^*^P = 0.042, ^*^P = 0.0166, ^*^P = 0.0182, *P* = 0.0556), **e** (from left to right, ^**^P = 0.0011, ^***^P = 0.0005), **f** (^***^P < 0.0001), **h** (^**^P = 0.0049), **j** (from left to right, ^*^P = 0.0487, ^**^P = 0.008, ^**^P = 0.0099, ^#^*P* = 0.0486). Source data are provided in Source Data file.

To determine whether the *Nrg1* enhancer is essential in hyperglycemic responses, we generated deletion mutants targeting the *Nrg1* enhancer using the CRISPR/Cas9 system. Targeted sequences within the *Nrg1* enhancer region were selected based on analysis of transcription factor-binding sites, including two partial Rbpj-binding sites inside the 250 bp enhancer, RBPJ, Jun proto-oncogene/Fos proto-oncogene (Ap1), and GATA4-binding sites^22^. The mutant cells were denoted as ΔE-250, ΔE-Rbpj, ΔE-Ap1, and ΔE-Gata4, respectively (Supplementary Fig. 7a). *Nrg1* levels in mutant and wild-type cells were measured following HG treatment. HG-induced *Nrg1* upregulation observed in wild-type cells was abrogated in ΔE-250 and ΔE-Rbpj cells (Fig. 5a). Although ΔE-Ap1 cells showed upregulation of *Nrg1* following HG treatment, *Nrg1* levels after 72 h of HG was still lower than that of WT, suggesting that AP1 also contributes to *Nrg1* overexpression upon HG treatment. This is consistent with previous reports that AP1 could drive transcriptional activity of the Nrg1 enhancer^5^. NRG1 protein levels were also consistently lower in ΔE-250 and ΔE-Rbpj cells treated with HG compared with wild-type cells (Fig. 5b), indicating that the *Nrg1* enhancer plays a crucial role in hyperglycemia-induced *Nrg1* expression and functions at least in part through Rbpj. DAPT-induced decreases in *Nrg1* levels observed in wild-type cells were not observed in ΔE-250 and ΔE-Rbpj cells (Supplementary Fig. 7b). Inhibition of intrinsic Notch signaling by DAPT was confirmed using NICD immunoblots (Supplementary Fig. 7c). In addition, deficiencies of *Nrg1* enhancer elements in ΔE-250 mutant cells resulted in loss of Nrg1 overexpression following TSA treatment (Supplementary Fig. 7d). Collectively, these results strongly indicate that Notch activation, which recruits Rbpj to the *Nrg1* enhancer, is required for hyperglycemia-induced *Nrg1* overexpression.

The viability of each mutant cell under HG and LG conditions was monitored for two days to determine the impact of *Nrg1* enhancer on the tumorigenic activities of Eo771 cells. Increased cell proliferation observed in response to HG in wild-type cells was significantly reduced in both ΔE-250 and ΔE-Rbpj cells (Fig. 5c). Basal cellular proliferation levels were also slightly attenuated in ΔE-250 and ΔE-Rbpj cells grown under LG conditions (Fig. 5c). Therefore, we validated ΔE-250 and ΔE-Rbpj tumor growth in vivo. We used a syngeneic tumor allograft model where the enhancer-edited Eo771 breast cancer cells are orthotopically injected into the euglycemic wild-type host. Because ΔE-250 and ΔE-Rbpj cells displayed decreased proliferation under HG conditions in vitro, we tested the tumorigenic capacity of the cancer cells under HG conditions. Consistent with in vitro data, tumor progression in mice allografts with either ΔE-250 or ΔE-Rbpj cancer cells was significantly attenuated compared with that in mice injected with wild-type control cells (Fig. 5d, e).

### Combination treatment using lapatinib and Notch inhibitor effectively ameliorates hyperglycemia-induced drug resistance to lapatinib

The NRG1/HER3 axis confers resistance to trastuzumab in HER2-positive breast cancer cells^23^. Moreover, compensatory activation of Notch during HER2-targeted therapy was implicated in drug resistance and tumor recurrence; thus, Notch inhibition was suggested to prevent HER2-targeted therapy^24^. Given that DAPT-induced Notch inactivation attenuated hyperglycemia-induced *Nrg1* overexpression, we hypothesized that the dual inhibition of NOTCH and HER2 could effectively reduce tumor burden, especially in breast cancer patients with hyperglycemia. Modest and short-term hyperglycemia was induced by multiple low doses of streptozotocin (STZ) injection to mice, and serum glucose levels were measured (Fig. 5f). The growth of Eo771 cells injected into mammary fat pads of mice was augmented by STZ, as determined by tumor volumes and weights (Fig. 5g, h). In modest and short-term hyperglycemic mice, the therapeutic efficacy of dual inhibition of NOTCH and HER2 pathways was assessed using lapatinib (100 mg/kg), an FDA approved drug that inhibits tyrosine kinase activity of HER2/EGFR, and a relatively low dose of DAPT (25 mg/kg). DAPT and lapatinib inhibited tumor growth in control mice (Fig. 5i, j). Individually, neither lapatinib nor DAPT affected tumor growth in STZ-induced modest-temporary hyperglycemic mice. However, the therapeutic efficacy was significantly improved when lapatinib and DAPT were combined, and this sensitizing effect was more evident in hyperglycemic mice than in control mice (Fig. 5i, j). Our results indicate that lapatinib resistance was augmented by hyperglycemia, and the impeded drug response was partially alleviated by combining lapatinib with DAPT, highlighting the potential value of the combined drug regimen as a therapeutic strategy for treating HER2-positive breast cancer patients with hyperglycemia.

### High *NRG1* levels are associated with poor outcomes in patients with HER2-positive breast cancer

To assess the prognostic value of Nrg1 in breast cancer, we analyzed whether *NRG1* is overexpressed in specific breast cancer subtypes. Human breast cancer cell lines with different receptor status, including SK-BR-3, MCF7, T47-D, BT-474, ZR75-30, MDA-MB-231, and BT-20, were analyzed. Most cells, excluding MDA-MD-231, showed upregulated *NRG1* expression up to five days after HG treatment (Supplementary Fig. 8a). Similar results were observed at the protein level; however, no changes were observed in BT-20 cells (Supplementary Fig. 8b). Importantly, the *NRG1* enhancer region was conserved across various species (Supplementary Fig. 8c). To check the chromatin status of the *NRG1* enhancer following stimulation with HG, ChIP-qPCR performed using various cancer cells revealed that the *NRG1* enhancer region was largely enriched by H3K27ac but not by H3K4me1 (Supplementary Fig. 8d). These results suggest that most breast cancer cells are responsible for HG-induced activation of the NRG1 enhancer, although *NRG1* overexpression in triple negative breast cancer cells such as MDA-MB-231 and BT-20 is less affected by HG.

**Fig. 6:**
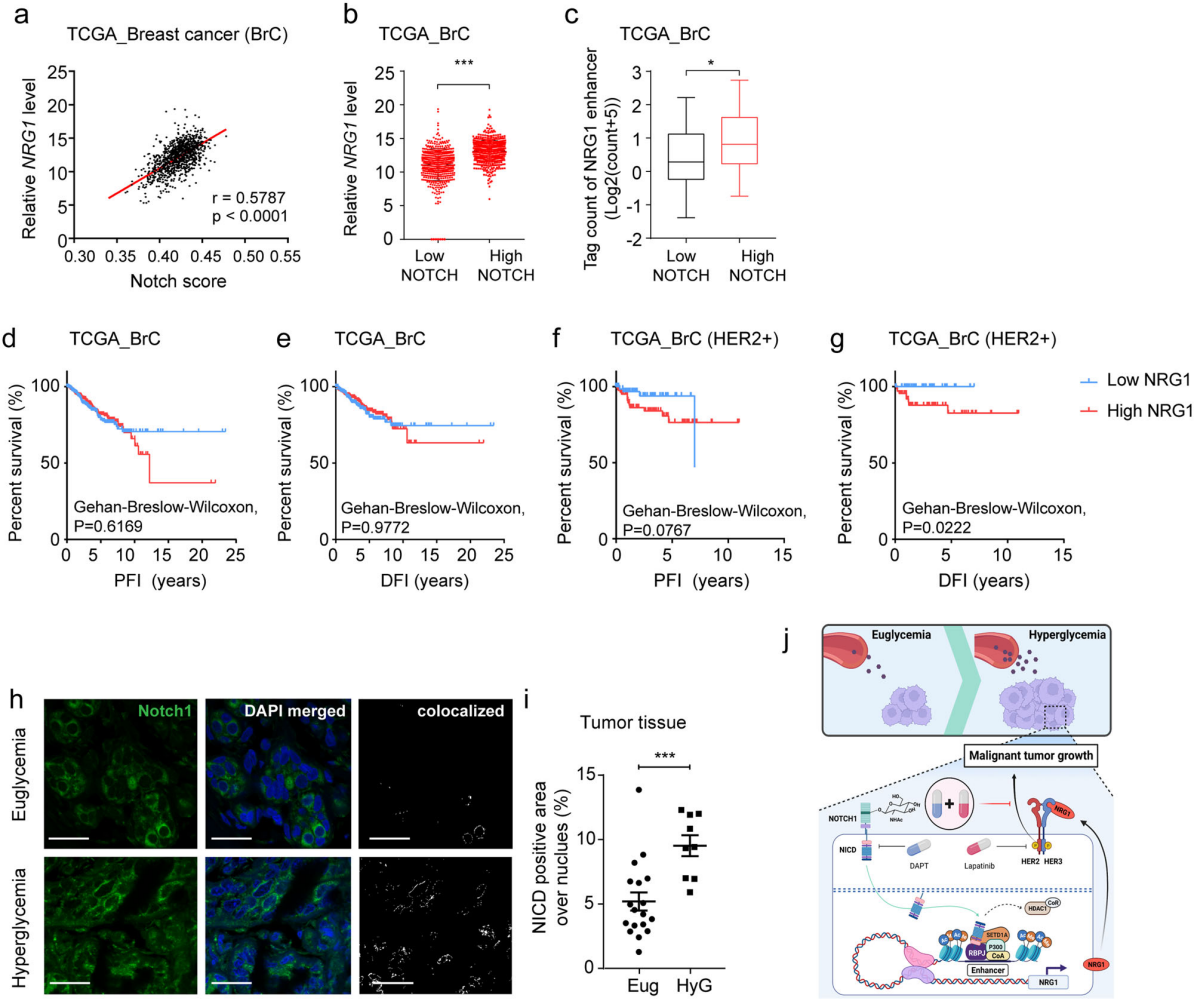
NRG1 predicts poor prognosis of HER2-positive breast cancer. **a–c** Analysis of breast cancer data obtained from TCGA. **a** Correlation between *NRG1* level and NOTCH activity in breast cancer patients (*n* = 1077) from TCGA database. **b** *NRG1* levels in high and low-NOTCH patient groups, stratified by median score (*n* = 538 each). **c** Open-chromatin accessibility of the *NRG1* enhancer regions in high and low-NOTCH patient groups from TCGA breast cancer cohort, stratified by median score (*n* = 37 each). **d, e** Kaplan–Meier survival analysis of PFI and DFI in low-*NRG1* (*n* = 538) and high-*NRG1* (*n* = 539) patient groups from TCGA breast cancer cohort. **f, g** Kaplan–Meier survival analysis of PFI and DFI in the low-*NRG1* (*n* = 70) and high-*NRG1* (*n* = 91) patient groups within the HER2-positive breast cancer cohort in TCGA. **h, i** Immunofluorescence staining of NOTCH1 and quantification of NOTCH1 signal co-localized with nuclei in breast tumor tissue samples from HER2-positive breast cancer patients (*n* = 18 for euglycemia, *n* = 9 for hyperglycemia) (scale bars; 20 μm). **j** Graphical summary of the study. Data are represented as mean ±SEM in **b, i** and as box and Whisker plot in **c** (box extends from the 25th to 75th percentiles, and whiskers ranging from minimum to maximum). Results shown in **a–i** are derived from single analysis (*n* = 1). Statistical significance was evaluated using two-tailed Pearson correlation test **a**, two-tailed Student’s *t*-test **b**, **c**, two-tailed Gehan‒Breslow‒Wilcoxon test **d–g**, and two-tailed Student’s *t*-test **i**. ^*^P < 0.05; ^**^P < 0.01; ^***^P < 0.001, **a** (^***^P < 0.0001), **b** (^***^P < 0.0001), **c** (^*^P = 0.0119), **d–g** (**d** to **g**, *P* = 0.6169, *P* = 0.9772, *P* = 0.0767, ^*^P = 0.0222), **i** (^***^P = 0.0009). Source data are provided in Source Data file.

To confirm the impact of NOTCH on *NRG1* levels, TCGA and ATAC-sequencing data were analyzed^25^. Since the Notch pathway was activated under hyperglycemic conditions, we categorized samples into low- and high-NOTCH groups and analyzed their enhancer accessibility and association with *NRG1* levels. *NRG1* levels were positively correlated with NOTCH activity in breast cancer patients (Fig. 6a). Patients with high-NOTCH scores had significantly higher *NRG1* expression than patients with low-NOTCH scores (Fig. 6b), regardless of HER2 status (Supplementary Fig. 9a, b). The chromatin of the *NRG1* enhancer region was also more accessible in the high-NOTCH group than in the low-NOTCH group, based on ATAC-sequencing results (Fig. 6c). To evaluate the prognostic value of *NRG1* levels in breast cancer, subjects from the TCGA database were classified into low- or high-NRG1 groups based on *NRG1* expression. Kaplan‒Meier survival analysis revealed no significant difference in overall survival (OS), progression-free interval (PFI) and disease-free interval (DFI) between these groups (Supplementary Fig. 9c and Fig. 6d, e). Since the NRG1-HER3 axis is implicated in resistance to HER2-targeted therapy, the impact of *NRG1* levels was investigated in HER2-positive breast cancer patients instead of the whole TCGA breast cancer cohort. The HER2-positive cohort was subdivided into low- and high-NRG1 groups based on their initial label, and subjected to Kaplan–Meier survival analysis, which revealed that *NRG1* expression significantly impacted DFI, with PFI reaching near statistical significance (*P* = 0.0767), where the high-NRG1 group exhibited worse prognosis (Fig. 6f, g). No significant difference was observed between HER2-negative groups (Supplementary Fig. 9d, e). Finally, we verified NOTCH activity in tissues from HER2-positive breast cancer patients with or without hyperglycemia. Although there were some confounding variables between the subjects, including the treatment status of HER2-targeted or hormonal therapy, cancer patients with hyperglycemic episodes had significantly higher NOTCH activity based on immunofluorescence staining of nuclear NOTCH1 (Fig. 6h, i). Collectively, our results indicated that high-NOTCH breast cancer patients had high *NRG1* levels, and elevated NOTCH activity is found in hyperglycemic breast cancer patients. We also highlighted the prognostic value of *NRG1*, as it seemed to be a good responder against HG-linked *NRG1* overexpression in HER2-positive versus HER2-negative patients.

## Discussion

Here, we showed that the *Nrg1* enhancer is activated by a hyperglycemic cue mediated by Notch-driven active histone modification following Nrg1 enhanceosome assembly. It has been reported that Notch activation promotes NRG1 expression through direct binding of NICD to the *Nrg1* promoter in melanoma^26^ or indirectly through the involvement of other mediators in cardiac tissues^27^. Beyond these regulatory mechanisms of Nrg1, our study showed that the NICD-RBPJ complex is assembled on the *Nrg1* enhancer and drives *Nrg1* overexpression upon HG stimulus. NRG1 overexpression results in activation of NRG1-HER3 signaling, leading to malignant cancer growth. We further showed that the oncogenic effect of NRG1 could be blocked by inhibiting both Notch and HER2 **(**Fig. 6j**)**.

Glucose is the primary energy source for multiple cells, and cells regulate glucose homeostasis through multiple glucose-sensing mechanisms^28^. One way of sensing glucose availability is via O-GlcNAc modification, wherein high glucose levels enhance HBP flow and subsequent O-GlcNAcylation of target proteins^11^. This modification regulates protein activities, allowing cells to adapt to environmental conditions^29^. In chronic hyperglycemia, several target proteins undergo aberrant O-glycosylation, including Sp1 and FoxO1^29^. O-GlcNAc of NOTCH1 was promoted by HG, which facilitated Notch activation and subsequent *Nrg1* overexpression. However, it remains unclear whether glucose triggered post-translational modifications (PTM) in other proteins, such as P300 and SETD1A, to facilitate the *Nrg1* enhancer recruitment. Although several studies reported that promoter binding of P300 is increased by HG stimulus, the detailed mechanism that links glucose and P300 recruitment is still poorly understood^30,31^. Beyond the PTM of NOTCH or other targets, tumorigenic signals could be attributed to direct involvement of excess glucose through activation of the *Nrg1* enhancer. As the epigenetic modifications rely on the metabolic status of cells using metabolites as cofactors or substrates^32^, hyperglycemia could contribute the hyperacetylation of the *Nrg1* enhancer. Acetyl-CoA abundance is regulated by glucose accessibility, and its production facilitates histone acetylation^33,34^. Thus, it is plausible to propose that hyperglycemia-induced reprogramming of glucose/acetyl-CoA metabolism could sustain tumor development^33^.

RBPJ is a statically DNA-bound protein independent of Notch activation; however, recent studies show that RBPJ can be dynamically recruited to target elements in a Notch-inducible manner^35,36^. A genome-wide ChIP-seq study identified a subset of dynamic Notch targets characterized by sensitive responses to Notch on/off status and lack of Notch-binding elements in promoter regions; instead, their transcriptional regulation was largely dictated by a distal enhancer enriched with H3K27ac^37^. In this study, Notch activation promoted the binding of NICD-RBPJ complex to the distal enhancer region of *Nrg1* in an HG-inducible manner, suggesting that *Nrg1* is a dynamic Notch target under the control of the enhancer. HG reduced the binding occupancy of HDAC1, a component of RBPJ co-repressor complex, as it was dissociated from the *Nrg1* enhancer. Inhibiting HDAC1 with TSA was sufficient to switch on *Nrg1* expression even under LG conditions. Thus, HDAC1 suppresses *Nrg1* expression under LG conditions, and its dissociation by HG-linked Notch activation seems to be crucial in shaping HG-inducible enhanceosome assembly.

The therapeutic potential of the metabolic status of breast cancer patients has been largely ignored, as therapeutic outcomes have been unsatisfactory. Approximately 35% of HER2-positive breast cancer patients initially responded to trastuzumab, of which only 30% exhibited no progression within a year^38^. One possible reason for the resistance to HER2-targeted therapy is hyperglycemia, which may arise during anticancer treatment or before cancer develops. The detrimental impact of diabetes on clinical outcomes of HER2-positive breast cancer treatment using trastuzumab has been reported^39,40^. Additionally, Notch signaling has recently been identified as critically important for trastuzumab resistance. Thus, a combination of a Notch antagonist and HER2-targeted drugs (trastuzumab, lapatinib) has been investigated as a therapeutic strategy to prevent drug resistance or cancer recurrence^24,41^. The role of Notch signaling in tumor progression has been extensively explored^42^, and numerous studies have focused on the potential of Notch inhibitors as cancer therapeutic drugs^43^. In clinical trials, several Notch inhibitors have been tested for their safety and therapeutic efficacy, including γ-secretase inhibitors (GSI), antibody-based drugs, and small molecules targeting the Notch-Rbpj complex^43,44^. Notch activation is also implicated in diabetes and its complications, and Notch-targeted therapy is proposed as a promising strategy for diabetic intervention^45,46^. Considering the applications of the Notch inhibitors in cancer and diabetes, Notch-targeting drugs are expected to be beneficial, especially in diabetic cancer patients. However, these drugs still lack detailed mechanisms and require further elucidation. Because Notch activity is elevated in HG-treated cancer cells and tissues from HER2-positive breast cancer patients with hyperglycemia, we postulated that hyperglycemia-driven NOTCH activation in breast cancer cells aggravated resistance to HER2-targeted therapy. Lapatinib resistance in hyperglycemic mice was reversed by combining lapatinib with a NOTCH antagonist. As the NRG1-HER3 axis contributes to resistance to HER2-targeted therapy^47^, blocking the Notch pathway and the subsequent NRG1 downregulation might account for the beneficial effect of lapatinib-DAPT combination treatment on hyperglycemia-driven drug resistance. These findings suggest that NRG1 mediates the crosstalk between HER2 and Notch pathway under hyperglycemic conditions, possibly by acting as a NOTCH target and a HER3 ligand.

There was no matching blood glucose or HbA1c data associated with the gene expression data in TCGA; hence, we postulated that elevated NOTCH activity might represent the patients’ hyperglycemic status. Although the diabetic status of breast cancer patients was unknown, we demonstrated that patients with high NOTCH activity had upregulated *NRG1* expression, regardless of breast cancer subtype, and had more accessible chromatin structure within the *NRG1* enhancer region. Additionally, *NRG1* overexpression predicted poor outcome in hyperglycemic HER2-positive cancer patients but not in HER2-negative patients. Due to a complexity of the diabetic pathophysiology, it is challenging to select proper hyperglycemic animal model; obesity-linked type 2 diabetes is considered as relevant model that recapitulates most of hyperglycemic patients. However, other confounding factors could influence tumor progression, including hyperinsulinemia, dyslipidemia, and inflammation, and thus make it difficult to distinguish the effect of hyperglycemia from that of other factors. This posed a limitation to the mouse model used in this study. To highlight the effect of hyperglycemia, we exposed mice to mild short-term hyperglycemia using low-dose STZ treatments. Therefore, additional mouse models that induce persistent and high hyperglycemia while minimizing inflammation may be considered for further studies.

In conclusion, although the metabolic state of patients is often overlooked, it should be taken into consideration to improve predicted responses and identify patients for whom drug combination therapy would be suitable. We propose that the diabetic status and NOTCH-NRG1-HER3 axis can be used as prognostic markers to predict responses to HER2-targeted therapy, offering a therapeutic strategy for treating breast cancer patients with diabetes.

## Methods

Our study was compliant with all relevant ethical regulations and the guidelines approved by the Ulsan National Institute of Science and Technology (UNIST). Patients were not monetarily compensated in this study.

### Cell cultures and hyperglycemic cell models

The murine breast cancer cell line Met1 was isolated from MMTV-PyMT mice, while Eo771 (CRL-3461) and 4T1 (CRL-2539) breast cancer cell lines were acquired from the American Type Culture Collection (ATCC). Human breast cancer cell lines ZR75-30 (CRL-1504), BT-474 (HTB-20), SK-BR-3 (HTB-30), MCF7 (HTB-22), MDA-MB-231 (CRM-HTB-26), BT-20 (HTB-19), and T47-D (HTB-133) were also acquired from ATCC. BT-20 cell line is under the list of known misidentified cell lines provided from the International Cell Line Authentication Committee (ICLAC). However, BT-20 cell was used as one of the triple negative breast cancer cells, which displayed limited responses to high glucose, and thus, it does not compromise our results. Cell culture medium was supplemented with 10% FBS and 1% penicillin/streptomycin, and cells were maintained in a 5% CO_2_ humidified incubator. To establish a cell line-based model system for studying hyperglycemia-driven breast cancer cells, a low glucose (1 g/L) adaptation period was applied. All the breast cancer cells were maintained in low glucose DMEM for at least 3 days and then transferred to high glucose (4.5 g/L) DMEM until the indicated time points.

### Plasmid and siRNA transfection

Plasmid vectors and small interfering RNAs (siRNAs) were transiently transfected into target cells using jet-OPTIMUS transfection reagent (Polyplus, #117-01) and G-Fectin (Genolution, Seoul, Korea), respectively, following the manufacturer’s instructions. Scrambled siRNAs were purchased from Genolution (Seoul, Korea). The siRNAs used in this study are listed in Supplementary Table 1.

### shRNA knockdown cell line

*P300*, *Cbp*, *Setd1A*, and *Nrg1*-targeting oligomers were annealed and cloned into pLKO.1 TRC vectors. H293T cells were transfected with VSVG, Δ8.9, and pLKO.1 vectors, and lentivirus-containing supernatant was collected twice and filtered with a 0.45 μm filter. Cancer cells were treated with lentiviral media and polybrene, and infected cells were selected by puromycin treatment. The sequences of hairpins used in this study are listed in Supplementary Table 2.

### Chemical reagents and antibodies

TSA and PUGNAc (Sigma, USA) were dissolved in DMSO to make stock solutions (500 µM and 50 mM, respectively) and then diluted to desired concentrations for in vitro assays. For osmotic control experiments, mannitol (Sigma, USA), and D-glucose (Amresco, USA) were dissolved in PBS (1 M) before use. The gamma-secretase inhibitor, DAPT (SelleckChem, USA), and the selective RTK inhibitor, lapatinib (TCI, Tokyo, Japan), were dissolved in DMSO to make 100 mM stock solutions and subsequently diluted to desired concentration for in vivo assays. STZ (Sigma, USA) was dissolved in 0.1 M citrate buffer (pH 4.5) just before injecting the animals. The primary antibodies used were against: H3K4me1 (Abcam, ab8895, 2 μg for ChIP), H3K27ac (Abcam, ab4729, 2 μg for ChIP), NRG1 (Abcam, ab27303, 1:800 dilution in western blot (WB)), NOTCH1 (Abcam, ab27526, 1:1,000 dilution in WB, 1:100 in ChIP, 1:100 in immunofluorescence), RBPJ (Abcam, ab25949, 1:1,000 dilution in WB, 2 μg in ChIP), O-GlcNAc (Abcam, ab2735, 1:1,000 dilution in WB, 1:200 in immunofluorescence), P300 (Abcam, ab275378, 1:1,000 dilution in WB, 1:100 in ChIP), CBP (CST, #7379, 1:1,000 dilution in WB, 1:100 in ChIP), HDAC1 (CST, #34589, 1:1,000 dilution in WB, 1:50 in ChIP), SETD1A (CST, #50805, 1:1,000 dilution in WB, 1:50 in ChIP), GAPDH (SCBT, sc-32233, 1:1,000 dilution in WB), Lamin A/C (SCBT, sc-376249, 1:1,000 dilution in WB), and β-Actin (SCBT, sc-47778, 1:1,000 dilution in WB). The secondary antibodies used are as follows; IR-dye 680 anti-mouse (Li-cor, P/N: 926-68070, 1:15,000 dilution in WB), IR-dye 800 anti-mouse (Li-cor, P/N: 926-32210, 1:15,000 dilution in WB), IR-dye 800 anti-rabbit (Li-cor, P/N: 926-32211, 1:15,000 dilution in WB), Alexa488 anti-rabbit (Invitrogen, A21206, 1:1,000 dilution in immunofluorescence), Alexa594 anti-mouse (Invitrogen, A11005, Lot 2043369, 1:2,000 dilution in immunofluorescence).

### Animal models

All animal experiments were conducted in accordance with protocols approved by the Institutional Animal Care and Use Committee (IACUC) of the Ulsan National Institute of Science and Technology (UNIST, UNISTIACUC-20-07). Mice were housed in air-filtered flow cabinets with a 12 h light cycle at 22 ± 2 °C and 55 ± 5% humidity, and allowed free access to water and food (Safe diet, Augy, France, composition; proteins % 20.80, lipids % 4.80, crude fiber % 3.70, total minerals % 5.60). For enhancer-deficient Eo771 tumor allografts, Eo771 cells were cultured in HG medium for one week. Wild-type *C57BL/6* *J* mice (eight-week-old) were obtained from Jackson Laboratory (stock# 000664), and then the cells were injected into the inguinal mammary fat pad of wild-type *C57bl/6* *J* male mice (0.8 × 10^6^ cells per mouse). Tumor burdens are allowed up to 5% of body weight of the mouse according to the guideline from IACUC of the UNIST, and thus tumors were harvested before exceeding the criteria (1.5 g of tumor weight for 30 g mice). For the diabetic breast cancer mouse model, STZ was freshly dissolved in 0.1 M citrate buffer (pH 4.5) and eight-week-old wild-type *C57bl/6* *J* female mice were given either vehicle (citrate buffer) or STZ (50 mg/kg) via intraperitoneal injections for five consecutive days. Fasting blood glucose levels were measured three days after the final STZ/vehicle injection, and Eo771 cells (0.8 × 10^6^ cells per mouse) were orthotopically injected into the inguinal mammary fat pad. Tumor growth was monitored, and tumors were harvested once tumor volumes reached approximately 800 mm^3^. For drug combination treatment, Eo771 cells (0.8 × 10^6^ cells per mouse) were orthotopically injected into inguinal mammary fat pads. Three days after cancer cell implantation, a single shot of either vehicle or STZ (110 mg/kg) was administered to the tumor-bearing mice. Fasting blood glucose levels were measured three days after STZ/vehicle administration, and the mice were injected with either vehicle or a combination of lapatinib and DAPT for six consecutive days. Lapatinib was freshly prepared in 30% polyethylene glycol (PEG) 400, 10% Tween 80, and 10% DMSO and administered orally (100 mg/kg), while DAPT was freshly prepared in corn oil (90%) and administered intraperitoneally (25 mg/kg). Tumors were harvested two days after treatment, before reaching 5% of body weight of the mice.

### Western blot assay

Cell lysates were prepared using NETN buffer (1% NP-40, 1 mM EDTA, 20 mM Tris–Cl, pH 7.5, 100 mM NaCl, 5 mM sodium pyrophosphate, 1 mM sodium orthovanadate, and 50 mM NaF), and proteins were quantified using the BCA protein assay kit (Thermo, MA, USA). Approximately 30 μg of proteins were resolved on 8% sodium dodecyl sulfate–polyacrylamide gels and transferred onto nitrocellulose membranes. The membranes were blocked with 5% skimmed milk for 45 min and then incubated overnight at 4 °C with primary antibodies. The membranes were then washed three times with TBST buffer and incubated with IR800 or IR680 dye-conjugated secondary antibodies for one hour. Signals were detected using an Odyssey CLx scanner (Li-COR Biosciences).

### RNA isolation and qPCR

Total RNA was extracted using TRIzol reagent (Invitrogen) following the manufacturer’s instructions. To synthesize complementary DNA (cDNA), 1 μg of total RNA was reverse-transcribed using MMLV-RT (Invitrogen), and qPCR was performed on the Quantstudio 5 system (Applied Biosystems, CA, USA) using SYBR green master mix (Enzynomics, Daejeon, Korea). The primers used for qPCR are listed in Supplementary Table 3.

### ChIP and Sequential ChIP

Chromatin was sheared using a truChIP® Chromatin Shearing Kit (PN520154, Covaris, MA, USA) following the manufacturer’s protocol, but with some modifications. Briefly, cells were fixed with 1% formaldehyde for 8 min at room temperature and then quenched with 0.125 M glycine. However, cells used for histone modification (H3K4me, H3K27ac) were not fixed. The cells were then washed, re-suspended in lysis buffer, and incubated on ice for 10 min. Nuclei were collected and chromatin shearing was performed using the Covaris S220 sonicator to obtain DNA fragments 200–500 bp in size. Sheared chromatin samples were diluted in ChIP dilution buffer (0.01% SDS, 1.1% Triton-X 100, 1.2 mM EDTA, 16.7 mM Tris-HCl, pH 8.1, and 167 mM NaCl) and precleared with protein A/G beads (Santa Cruz, sc-2003), rabbit serum, and salmon sperm DNA at 4 °C for 1 h. Precleared samples were incubated overnight at 4 °C with specific antibodies and then precipitated with protein A/G bead at 4 °C for 1 h. The immunoprecipitated DNA complex was sequentially washed with a low salt concentration buffer (0.1% SDS, 1% Triton-X 100, 20 mM Tris-HCl pH 8.1, 2 mM EDTA, and 10 mM NaCl), high salt concentration buffer (0.1% SDS, 1% Triton-X 100, 20 mM Tris-HCl, pH 8.1, 2 mM EDTA, and 500 mM NaCl), LiCl buffer (0.25 M LiCl, 1% NP-40, 1% deoxycholic acid, 1 mM EDTA, and 10 mM Tris-HCl, pH 8.1), and twice with TE buffer (10 mM Tris-HCl, pH 8.0 and 1 mM EDTA). Samples were eluted using ChIP elution buffer (1% SDS and 100 mM NaHCO_3_) at 25 °C for 30 min. For sequential ChIP assay, samples were eluted using re-ChIP elution buffer (15 mM DTT and 10 mM Tris pH 8.0, 2% SDS) at 37 °C for 30 min and subsequently diluted 1/20 in ChIP dilution buffer. Second antibody incubation, precipitation, sequential washes and elution steps were repeated. Eluted samples were incubated with RNase A and proteinase K, and then reverse cross-linked overnight at 65 °C. DNA samples were purified using a column-based kit (DN10200, Bionics, Seoul, Korea) and analyzed using qPCR. Primers used for ChIP-qPCR are listed in Supplementary Table 4.

### Chromatin accessibility assay

A chromatin accessibility kit was purchased from Abcam (ab185901), and the assay was performed according to the manufacturer’s instructions. Briefly, chromatin was isolated from LG or HG treated cells (2 × 10^6^) and digested with nuclease mix. DNA samples were purified and analyzed by qPCR. Primer sequences used for analyzing *Nrg1* enhancer and promoter are listed in Supplementary Table 5.

### Immunofluorescence staining

Met1 and 4T1 cancer cells were seeded overnight on coverslips. After HG treatment, cells were washed with PBS and fixed with 4% paraformaldehyde in PBS for 30 min. After rinsing, fixed cells were blocked with NH_4_Cl for 5 min, incubated overnight at 4 °C with primary antibodies against NOTCH1 and O-GlcNAc, and then with secondary antibodies labeled with Alexa488 or 594 for 1 h at room temperature. DAPI was added as a co-stain, and images were acquired using an Olympus Fv1000 confocal microscope.

### Viability assay

Breast cancer cells were seeded overnight in 96-well plates (2000 cells/well) and then treated with either siRNA or DAPT. The cells were then incubated in Incucyte Zoom system (Essen bioscience) for two days. Cellular proliferation was monitored by taking images every two hours. Confluency was measured using Incucyte software (Sartorius, Göttingen, Germany).

### Analysis of nuclear and cytoplasmic fractions

Cytoplasmic and nuclear extracts were prepared using a two-step hypotonic and high salt concentration buffer protocol. Harvested cells were washed with cold PBS and incubated on ice with hypotonic buffer (20 mM HEPES pH 7.9, 1 mM EDTA, 1 mM EGTA) for 20 min. The crude extracts were centrifuged at 14,000 *g* and 4 °C for 5 min, and cytoplasmic extracts were collected in the supernatant. The remaining nuclear pellets were re-suspended in a high salt concentration buffer (420 mM NaCl, 20 mM HEPES pH 7.9, 1 mM EDTA, 1 mM EGTA, 20% glycerol) and homogenized in an ice-cold dounce homogenizer. After 20 min on ice, the extracts were centrifuged at 15,000 × *g* and 4 °C for 20 min and nuclear extracts harvested in the supernatant.

### Luciferase assay

The *Nrg1* enhancer or promoter region was inserted into a pGL4.23 reporter vector (Promega, Madison, WI, USA) while Rbpj cDNA was inserted into pCMV6–Myc/Flag (Origene). Met1 cancer cells were transfected with control or the *Nrg1* enhancer reporter together with RBPJ expressing vectors. In addition, the control reporter, *Nrg1* enhancer, *Nrg1* promoter, or both reporter vectors were transfected into Met1 or 4T1 breast cancer cells, which were then treated with LG or HG. A Renilla luciferase construct was used to normalize transfection controls. Cell lysates were harvested, and luciferase activity was measured using the luciferase assay system (Promega), according to the manufacturer’s protocol.

### CRISPR/Cas9 genome editing

CRISPR/Cas9 genome editing was used to delete parts of *Nrg1* enhancer elements in Eo771 breast cancer cells using the lentivirus delivery system. Single guide RNAs (sgRNAs) targeting Rbpj, Ap1, and Gata4-binding motifs, as well as the whole *Nrg1* enhancer region were designed using the Benchling online tool (https://www.benchling.com/) (Supplementary Table 6). sgRNA pairs were annealed and inserted into the LentiCRISPRv2 plasmid vector. To produce lentiviruses, LentiCRISPRv2 constructs were co-transfected with VSVG and Δ8.9 enveloping/packaging vectors into H293T cells, and the conditioned medium was collected. Eo771 breast cancer cells were transduced with the conditioned medium containing lentiviral particles targeting each binding motif and the enhancer region, as well as a non-target sequence in the wild-type control. Transduced cells were selected using puromycin and single colony isolation. Standard PCR and commercial DNA sequencing were performed to validate target sequence deletions. We selected clones with the intended modifications in their target sequences: wild-type (Non-target) control, ΔE-250, ΔE-Rbpj, ΔE-Ap1, and ΔE-Gata4 (Supplementary Fig. 6a).

### Analysis of clinical data from TCGA

Survival annotations and RNA sequencing data of 1,077 breast cancer patients were downloaded from TCGA. To analyze gene expression profiles, FPKM counts were normalized through Log2 transformation. A hallmark Notch signaling gene set (M5903 from GSEA) was used to define the NOTCH score that was used to categorize TCGA cohort based on NOTCH activity. For each patient, the score was determined using the singscore method implemented in the R software singscore_1.8.0 (R version 4.0.2). The scores were then stratified based on the median value. To analyze open-chromatin accessibility in the *Nrg1* enhancer region, a subset of patients with ATAC-Seq data (*n* = 74) identified in previous reports^25^ were stratified based on median NOTCH activity (*n* = 37 per group). To analyze survival, breast cancer patients were initially divided into low- or high-NRG1 groups based initially on *NRG1* levels and then on HER2-status in TCGA. Individuals were classified into either HER2-positive (*n* = 161) or HER2-negative (*n* = 916) groups. Kaplan‒Meier curves were generated for both HER2-positive and HER2-negative groups using survival parameters (PFI and DFI) obtained from previously published data^48^. Statistical significance was calculated using Gehan-Breslow-Wilcoxon test implemented in GraphPad Prism 7.

### Bisulfite sequencing

Genomic DNA (gDNA) was extracted from Met1, 4T1, and Eo771 cells grown under LG and HG conditions. Bisulfite conversions of gDNA were performed using the EpiTect bisulfite kit (Qiagen, #59104) according to the manufacturer’s protocol. Methylation-specific primers were designed using MethPrimer (https://www.urogene.org/methprimer/) and converted gDNA was amplified using Platinum Taq polymerase (Thermo Scientific, #10966). PCR amplicons were cloned into pGEM T easy vector (Promega) and mapped via commercial sequencing. The primers used for methylation-specific PCR are listed in Supplementary Table 7.

### Pull down assay and mass spectrometry

Part of the *Nrg1* enhancer was amplified as an oligonucleotide bait for enhancer-binding proteins using biotinylated primers (Supplementary Table 7) synthesized by Cosmo Genetech (Seoul, Korea). Nuclear extracts from Met1 cancer cells were prepared in hypertonic and high salt concentration buffers and then incubated overnight at 4 °C with the enhancer baits and poly dI-dC (Sigma, P4929). Baits and nuclear protein complexes were precipitated using streptavidin-coated beads (Thermo Scientific, #20349) and eluted using Laemmli sample buffer. Each eluted sample (10%) was separated by SDS-PAGE and analyzed by silver staining (Sigma) according to the manufacturer’s protocol. To identify candidate proteins, the remaining portion of the eluted sample was separated by 10% SDS-PAGE until the samples covered 2 cm on the gel. The gels were divided into six slices and then subjected to in-gel tryptic digestion. The resulting tryptic peptides were analyzed using LC-MS/MS on the Orbitrap ELITE mass spectrometer (Thermo Fisher Scientific, MA, USA) equipped with a nanoelectrospray ion source. To separate peptides, we used a C18 reverse-phase HPLC column (250 mm × 75 μm ID) with a 2.4–35% acetonitrile/0.1% formic acid gradient at a flow rate of 300 nL/min for 120 min. For MS/MS analysis, the precursor ion scan MS spectra (m/z 400–2000) was acquired in an Orbitrap at a resolution of 60,000 at m/z 400 with an internal lock mass. Twenty ions with the highest intensities were isolated and fragmented by collision-induced dissociation (CID).

### Processing LC-MS/MS data

MS/MS samples were analyzed on the Sequest Sorcerer platform (Sagen-N Research, San Jose, CA). Sequest was set up to interrogate the *Mus musculus* protein database (17,278 entries, UniProt, GCA_000001635.8 from Ensembl (http://www.uniprot.org/)) that includes frequently observed contaminants, assuming trypsin is used. Sequest selected proteins with fragment ion mass tolerance of 1.00 Da and a parent ion tolerance of 10.0 ppm. Cysteine carbamidomethyl and methionine oxidation were specified as fixed and variable modifications, respectively. Scaffold v5.0.1 (Proteome Software Inc., Portland, OR, USA) was used to validate MS/MS-based peptide and protein identities. Peptide identities were accepted if they had >94.0% probability of achieving <1.0% FDR based on the Peptide Prophet algorithm with Scaffold delta-mass correction. Protein identities were accepted if they had >99.0% probability of achieving <1.0% FDR and contained at least two identified peptides. Protein probabilities were assigned using the Protein Prophet algorithm v5.0^49^. Proteins with similar peptides that could not be differentiated based on MS/MS analysis alone were grouped to satisfy the principles of parsimony.

### Analysis of human breast cancer tissues

HER2-positive human breast cancer samples were obtained with approval from the Seoul National University Hospital Ethical Committee (approval number: 2111-069-1271) and informed consent was waived considering the nature of the study (retrospective observation and use of de-identified tissues). Moreover, several patients had finished their follow-up or had been deceased. Age of all the patients is over 20, and all patients are female (*n* = 27) (Supplementary Table 8). Based on glycemic records of the breast cancer patients, subjects with repeated blood glucose levels greater than 110 mg/dL were categorized into hyperglycemic (*n* = 9) or euglycemic (*n* = 18) groups. Formalin-fixed breast cancer tissues were embedded in paraffin, and section slices were generated. Tissue sections were deparaffinized and rehydrated in serial xylene and ethanol washes (100, 90, 80%) and then incubated with antigens in citrate buffer (pH 6) in a pressure cooker before being blocked with 5% BSA. The samples were subsequently incubated overnight at 4 °C with primary antibodies against NOTCH1, and then with Alexa488-labeled secondary antibodies for 1 h at room temperature. DAPI was used as a co-stain, and images were acquired using an Olympus Fv1000 confocal microscope.

### Statistics and reproducibility

Statistical analyses were conducted using GraphPad Prism 7.0. Details of the analyses are described within the figure legends. At least three independent sets of western blot and qPCR analysis were performed to ensure reproducibility, and representative data are shown. For quantification of NICD immunofluorescence in tumor sections from human breast cancer patients, at least nine independent subjects were analyzed (*n* = 18 for euglycemic group, *n* = 9 for hyperglycemic group). For quantification of double immunofluorescence of NOTCH1 and O-GlcNAc in Met1 cancer cells, at least five independent biological replicates were analyzed (*n* = 6 for LG, and *n* = 5 for HG).

### Reporting summary

Further information on research design is available in the Nature Portfolio Reporting Summary linked to this article.

## Supplementary information

Supplementary information

Reporting Summary

Description of Additional Supplementary Files

Supplementary Data 1

## Data Availability

Survival annotations and gene expression data of breast cancer patients could be obtained from TCGA database (TCGA-BRCA project, dbGaPaccessionphs000178), which is freely available and accessible at https://portal.gdc.cancer.gov/projects/TCGA-BRCA. Survival annotations also could be accessible at 10.1016/j.cell.2018.02.052. The Nrg1 enhanceosome proteomics data generated in this study have been deposited to the ProteomeXchange Consortium via the PRIDE partner repository with the dataset identifier PXD038668 and 10.6019/PXD038. List of the Nrg1 enhancer binding proteins generated from the proteomics data is provided in the Supplementary data 1. Source data are provided with this paper. Datasets generated from current study are available from the corresponding author. Source data are provided with this paper.

## Source data

Source Data
