# Supplementary material for: Epigenetic regulation of Neuregulin 1 promotes breast cancer progression associated to hyperglycemia
Source: Nat Commun. 2023 Jan 27;14:439. doi: 10.1038/s41467-023-36179-8 (PMC9883495; doi:10.1038/s41467-023-36179-8)
Supplement: Supplementary file 1 — Supplementary information [file 41467_2023_36179_MOESM1_ESM.pdf]

Supplementary information

Epigenetic regulation of Neuregulin 1 promotes breast cancer progression associated to hyperglycemia

Changhu Lee[1], Min Kim[1], Chanho Park[1], Woobeen Jo[1], Jeong Kon Seo[2], Sahee Kim[1], Jiyoung Oh[1], Chu-Sook Kim[1], Han Suk Ryu[3], Kyung-Hun Lee[4] and Jiyoung Park[1*]

[1]Department of Biological Sciences, College of Information and Bioengineering, Ulsan National Institute of Science and Technology, Ulsan 44919, Republic of Korea

[2]UNIST Central Research Facility, Ulsan National Institute of Science and Technology, Ulsan 44919, Republic of Korea

[3]Department of Pathology, Seoul National University Hospital, Seoul National University College of Medicine, Seoul, Republic of Korea

[4]Department of Internal Medicine, Seoul National University Hospital, Cancer Research Institute, Seoul National University, Seoul, Republic of Korea

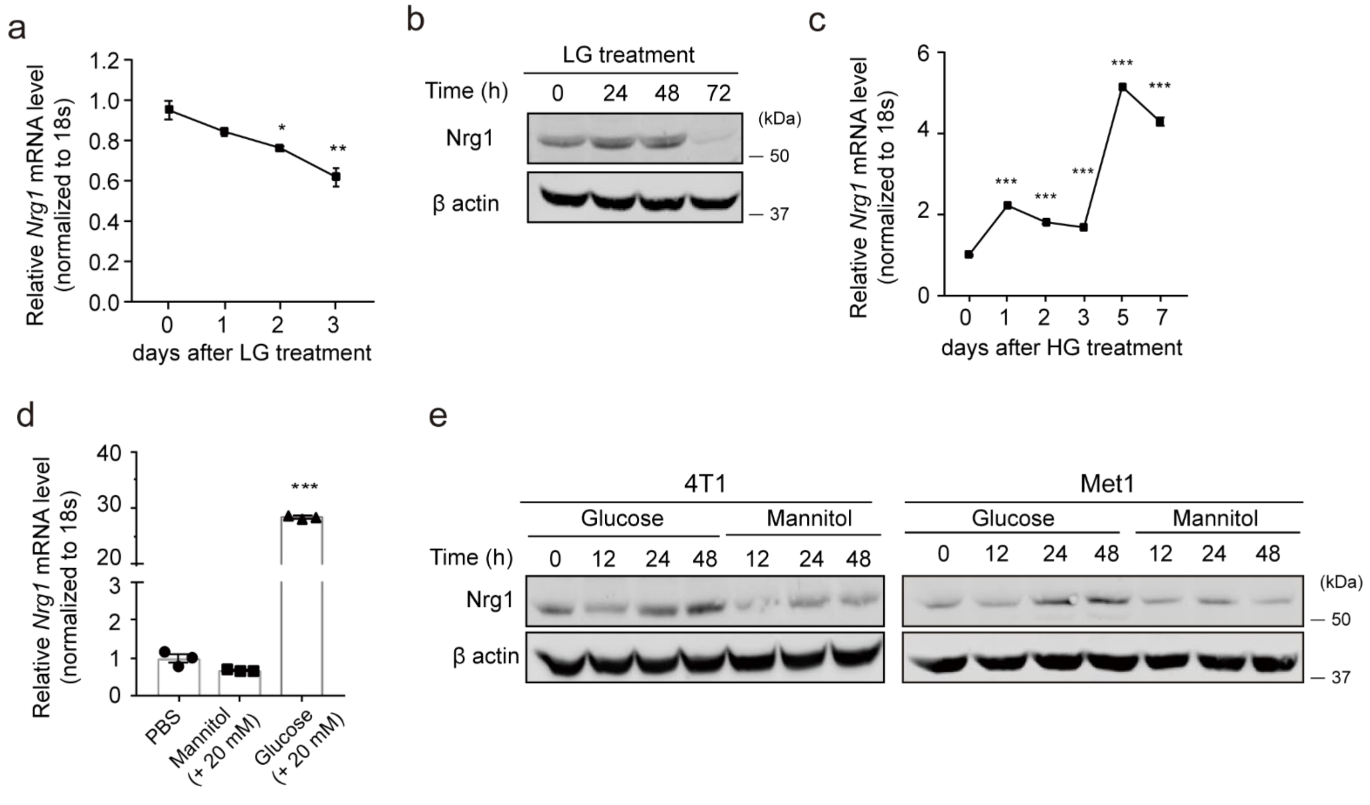

Supplementary Figure 1. Establishment of hyperglycemia system *in vitro*. To establish cancer cells into euglycemia-mimicking condition, glucose concentration of cell culture media was changed from 25mM (HG) to 5.5mM (LG) in 4T1 breast cancer cells up to 3days. **(a)** Both mRNA and **(b)** protein level of Nrg1 were diminished to basal level after 3 days of LG treatment. **(c)** LG-adapted 4T1 cells was returned to HG condition and cellular response to HG was confirmed by robustly elevated *Nrg1* level up to 7 days. Those cell culture models were further used in our *in vitro* hyperglycemic cancer cell study. **(d-e)** 4T1 cancer cells were treated with either 20mM of mannitol or glucose. (d) Both mRNA and (e) protein levels of the Nrg1 were elevated by glucose treatment only, but not by mannitol treatment, suggesting that HG is a main contributor of Nrg1 expression, rather than osmotic pressure. Data represented mean ±SEM of technical replicates in **a**, **c-d**, and n = 3 independent experiments were performed. Statistical significances were evaluated by *one-way ANOVA* followed by Dunnett's **a**, **c**, and Tukey's **d** post hoc test. (*$p < 0.05$; **$p < 0.01$; ***$p < 0.001$), **a** (*P=0.0458, **P=0.0064), **c** (all P<0.0001), **d** (***P<0.0001). Source data are provided in Source Data file.

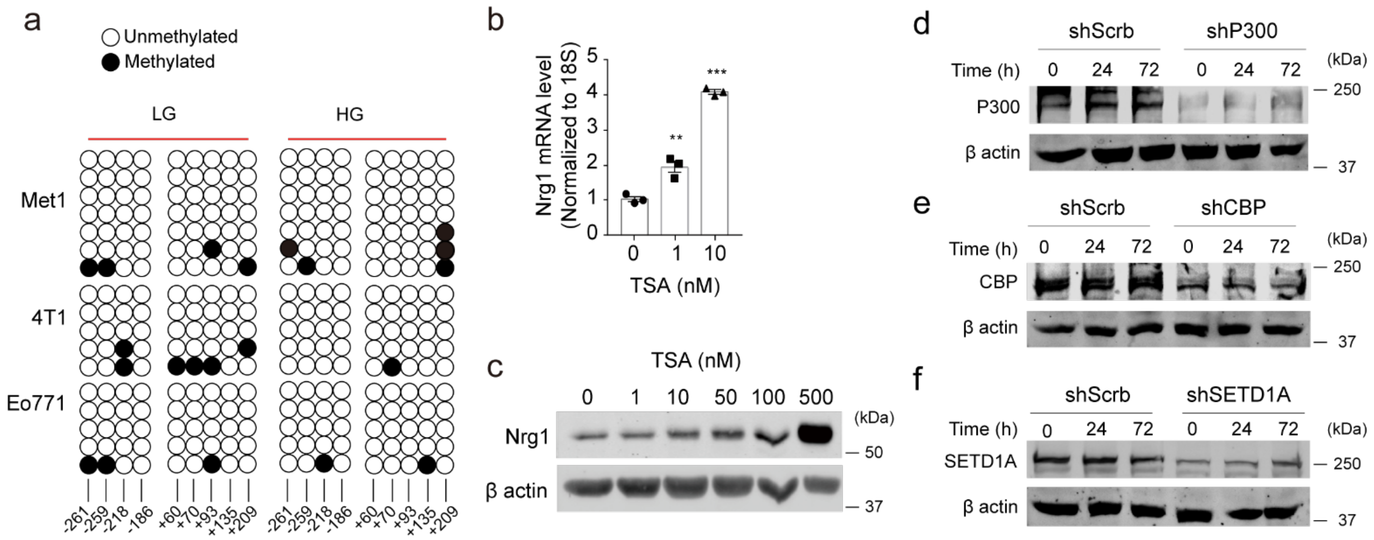

Supplementary Figure 2. Epigenetic regulation of the Nrg1 in the breast cancer. (a) Bisulphite sequencing was performed to analyze DNA methylation status of the Nrg1 enhancer in Met1, 4T1, and Eo771 cells from either LG or HG condition, revealing that most of CpGs were unmethylated all along the enhancer region. Each column represented CpG sites within the *Nrg1* enhancer region and each row represented independent clones (Closed circles; methylated CpGs, Open circles; unmethylated CpGs) **(b)** Both mRNA and **(c)** protein level of Nrg1 were upregulated in 4T1 cells treated with increasing dose of TSA under LG condition, suggesting that H3K27ac contributes to Nrg1 overexpression. **(d-f)** P300, Cbp, or Setd1A was depleted in the Met1 cells by lentiviral shRNA infection, and treated with HG for 72hours. Silencing of each targets including (d) P300, (e) Cbp, and (f) Setd1A was verified by western blot analysis. Data represented mean ±SEM of technical replicates in **b** and n = 3 independent experiments were performed. Statistical significances were evaluated by *one-way ANOVA* followed by Tukey's **b** post hoc test, (*p < 0.05; **p < 0.01; ***p < 0.001), **b** (**P=0.0024, ***p<0.0001). Source data are provided in Source Data file.

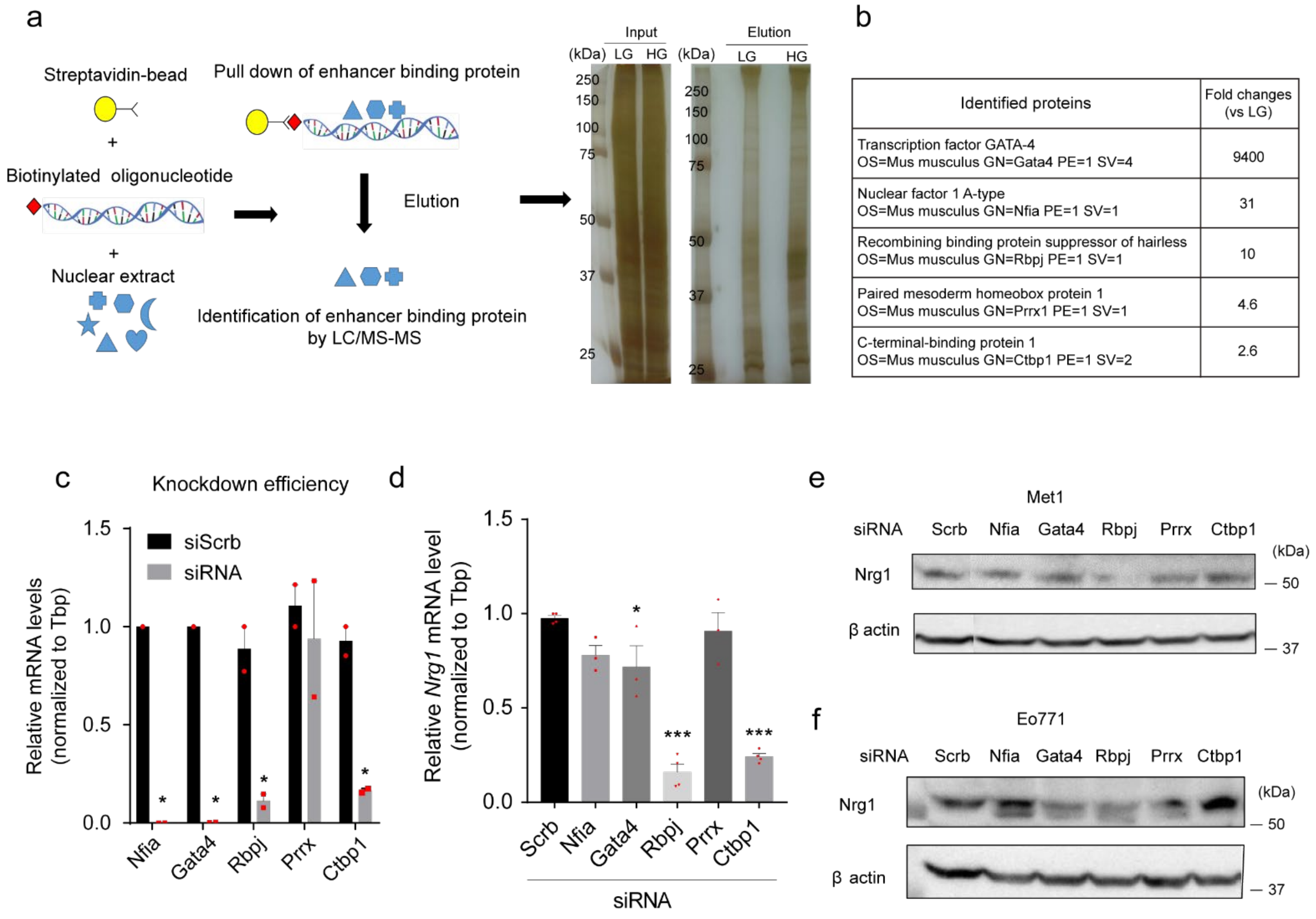

Supplementary Figure 3. Identification of Nrg1 enhancer binding proteins. (a) Experimental scheme for enhancer-baited pulldown assay was graphically described. Eluted proteins were analyzed by silver staining and identified by mass-spec proteomics approach. **(b)** Thousands of transcription factors were found to be more occupied in the enhancer-bait under HG condition, and whole list of identified proteins were listed in supplementary file. Among the candidates, 5 proteins were represented: Gata4, Nfia, Rbpj, Prrx1, and Ctbp1. **(c-f)** The 5 chosen candidates (Gata4, Nfia, Rbpj, Prrx1, and Ctbp1) were further tested whether they could regulate Nrg1 expression. Met1 or Eo771 cancer cells were treated with HG together with siRNA against the selected targets. (c) Knock-down efficiency of candidates were validated by mRNA level, and (d) *Nrg1* level was found to be reduced by transfection of the siRNA against Gata4, Rbpj and Ctbp1. (e-f) Protein levels of NRG1 were consistently decreased by siRNA against Rbpj in both Met1 and Eo771 cells, and thus we selected Rbpj as putative key regulator of the Nrg1 enhancer. Data represented mean ±SEM of technical replicates in **c, d** and n = 3 independent experiments were performed. Statistical significances were evaluated by *two-way ANOVA* followed by Bonferroni's **c** post hoc test, and *one-way*

ANOVA followed by Dunnett's **d** post hoc test. *$p < 0.05$; **$p < 0.01$; ***$p < 0.001$, **c** (from left to right, *$P=0.0074$, *$P=0.0075$, *$P=0.0102$, *$P=0.0112$), **d** (from left to right, *$P=0.0283$, ***$P<0.0001$, ***$P<0.0001$). Source data are provided in Source Data file.

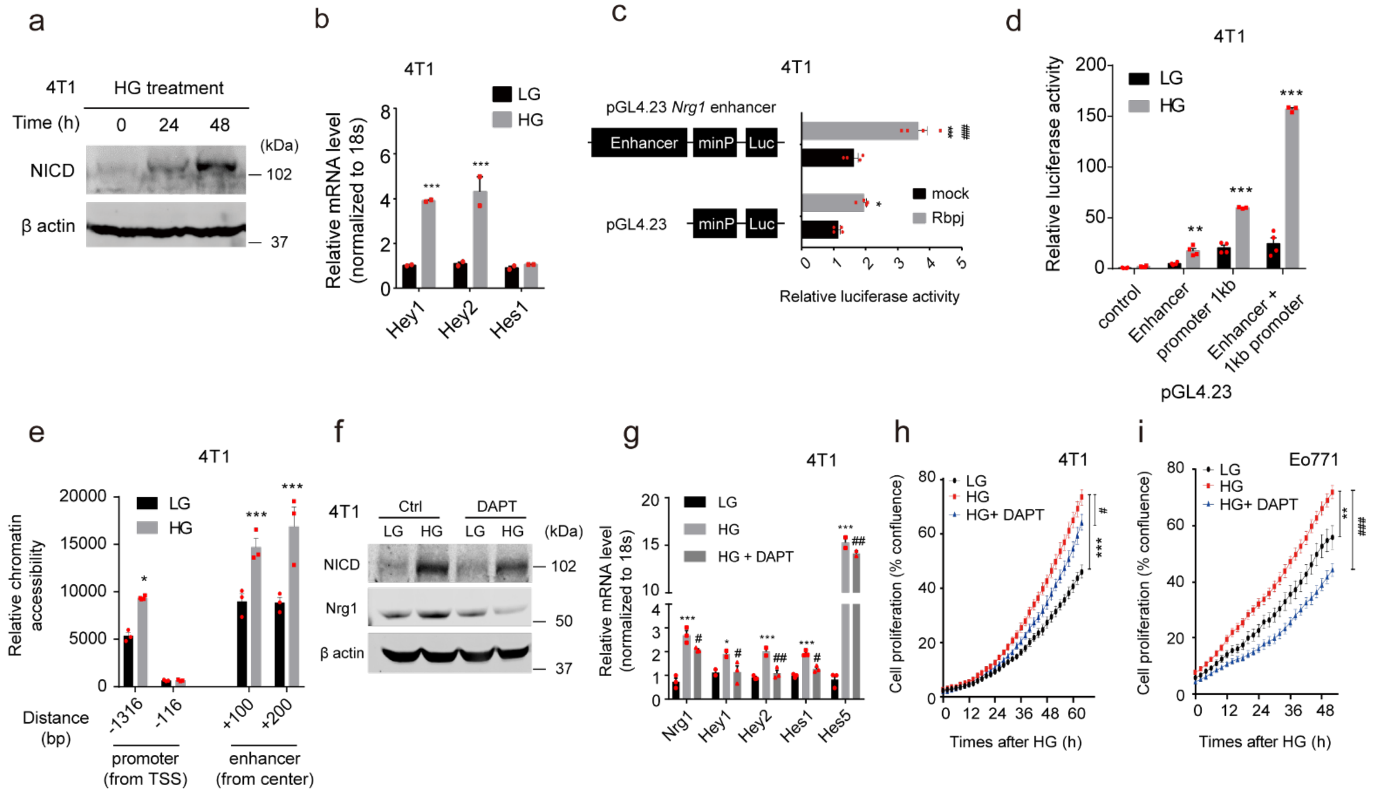

Supplementary Figure 4. Activation of Notch pathway under HG condition. (a-b) 4T1 cancer cell was treated with HG for 2 days. (a) NICD level and (b) mRNA levels of Notch target genes were elevated by HG treatment, suggesting that HG resulted in Notch activation. **(c)** Reporter constructs (pGL4.23) containing the Nrg1 enhancer were schematically represented, and 4T1 cells were transfected with indicated vectors. Luciferase activity was elevated by RBPJ, and it was further augmented in the presence of *Nrg1* enhancer, suggesting that RBPJ could drive activation of the *Nrg1* enhancer. **(d)** 4T1 cancer cells were transfected with the pGL4.23 reporter vectors containing control, Nrg1 enhancer, promoter, or enhancer with promoter and their reporter activity was found to be increased by HG treatment in enhancer or promoter element. Reporter activity of the enhancer with promoter was synergistically increased by combination of the elements. (e) Chromatin accessibility of the Nrg1 promoter (-1316, -116bp from TSS) and enhancer region (+100, +200bp from center of the enhancer) were analyzed by qPCR in the 4T1 cells treated with LG or HG. HG treatment elevated the chromatin accessibility of the Nrg1 promoter (-1316) and enhancer region (+100, +200bp). **(f-i)** To manipulate Notch pathway, either vehicle or DAPT were treated to the 4T1 cancer cells from LG or HG conditions. (f) HG treatment elevated protein levels of NRG1 and NICD, but they were abrogated by DAPT treatment. (g) Similar result was observed in mRNA level of Notch targets and *Nrg1* by DAPT treatment, and (h-i) HG-driven cellular proliferations were attenuated by

DAPT treatment in 4T1 and Eo771 cancer cells, suggesting that Notch signaling pathway is crucial in regulating HG-induced Nrg1 overexpression, which consequently support tumor growth. Data represented mean ± SEM of technical replicates in **b-e, g,** and biological replicates in **h-i**, and n = 3 independent experiments were performed. Statistical significances were evaluated by *two-way ANOVA* followed by Bonferroni's **b**, **d**, **e** and Tukey's **c**, **g**, **h**, **i** post hoc test. *p < 0.05; **p < 0.01; ***p < 0.001 for group1 versus indicated group and #p < 0.05; ##p < 0.01; ###p < 0.001 for column2 versus indicated column, **b** (from left to right, ***P=0.0009, ***P=0.0005), **c** (*P=0.0292, ***P<0.0001, ###P<0.0001), **d** (from left to right, **P=0.0073, ***P<0.0001, ***P<0.0001), **e** (from left to right, *P=0.0103, ***P=0.0008, ***P<0.0001), **g** (from left to right, ***P<0.0001, #P=0.0357, *P=0.0301, #P=0.0194, ***P=0.0009, ##P=0.0050, ***P=0.0014, #P=0.0257, ***P<0.0001, ##P=0.0015), **h** (***P<0.0001, #P=0.0373), **i** (**P=0.0048, ###P<0.0001). Source data are provided in Source Data file.

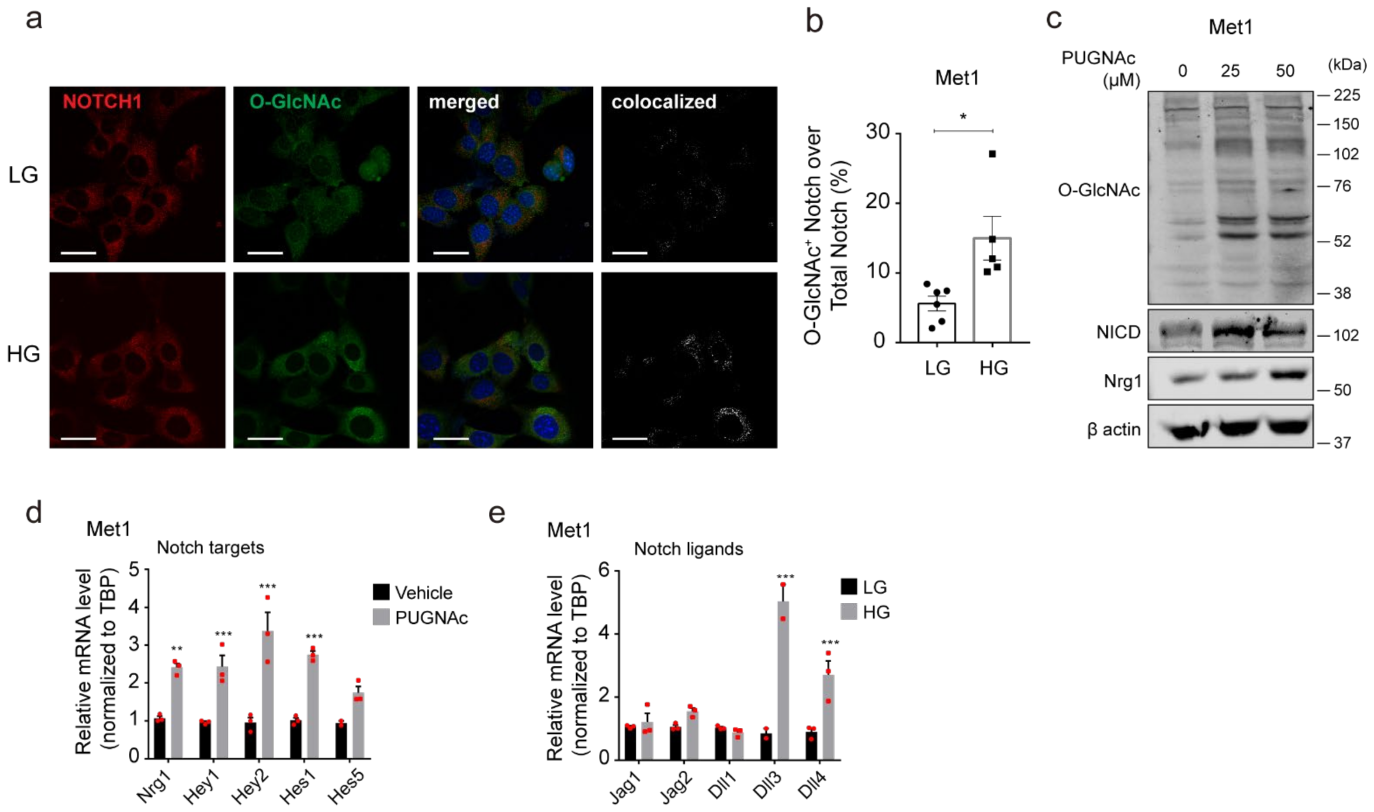

Supplementary Figure 5. HG induced Notch glycosylation and Nrg1 overexpression. (a-b) Double immunofluorescence images of Notch1 and O-GlcNAc were represented (scale bars; 20μm), and O-Glycosylated NOTCH1 signals were elevated by HG treatment in Met1 cells, which promotes ligand driven Notch activation. **(c-d)** To induce global increase of O-GlcNAc, increasing dose of PUGNAc was treated to Met1 cancer cells for 1 days under LG condition, and it was found that (c) protein level of NICD, NRG1 and O-glycosylated targets were elevated by PUGNAc treatment. (d) PUGNAc treatment led to increase in mRNA levels of *Nrg1* and Notch targets, suggesting that O-GlcNAcylation of Notch1 could switch on target gene expression and Nrg1 overexpression. **(e)** HG treatment elevated mRNA levels of Notch ligands in Met1 cells, suggesting that HG-derived Notch activation is partially due to upregulation of Notch ligand expressions. Data are represented as mean ±SEM (from technical replicates in **d-e,** and biological replicates in **b**). Results shown in **d-e** are representative of three independent experiments. Statistical significances were evaluated by two-tailed students' t-test **b** (*P=0.0133), and *two-way ANOVA* followed by Bonferroni's **d** (from left to right, **P=0.0010, ***P=0.0004, ***P<0.0001, ***P<0.0001), **e** (from left to right, ***P<0.0001, ***P<0.0001) post hoc test. (*p < 0.05; **p < 0.01; ***p < 0.001). Source data are provided in Source Data file.

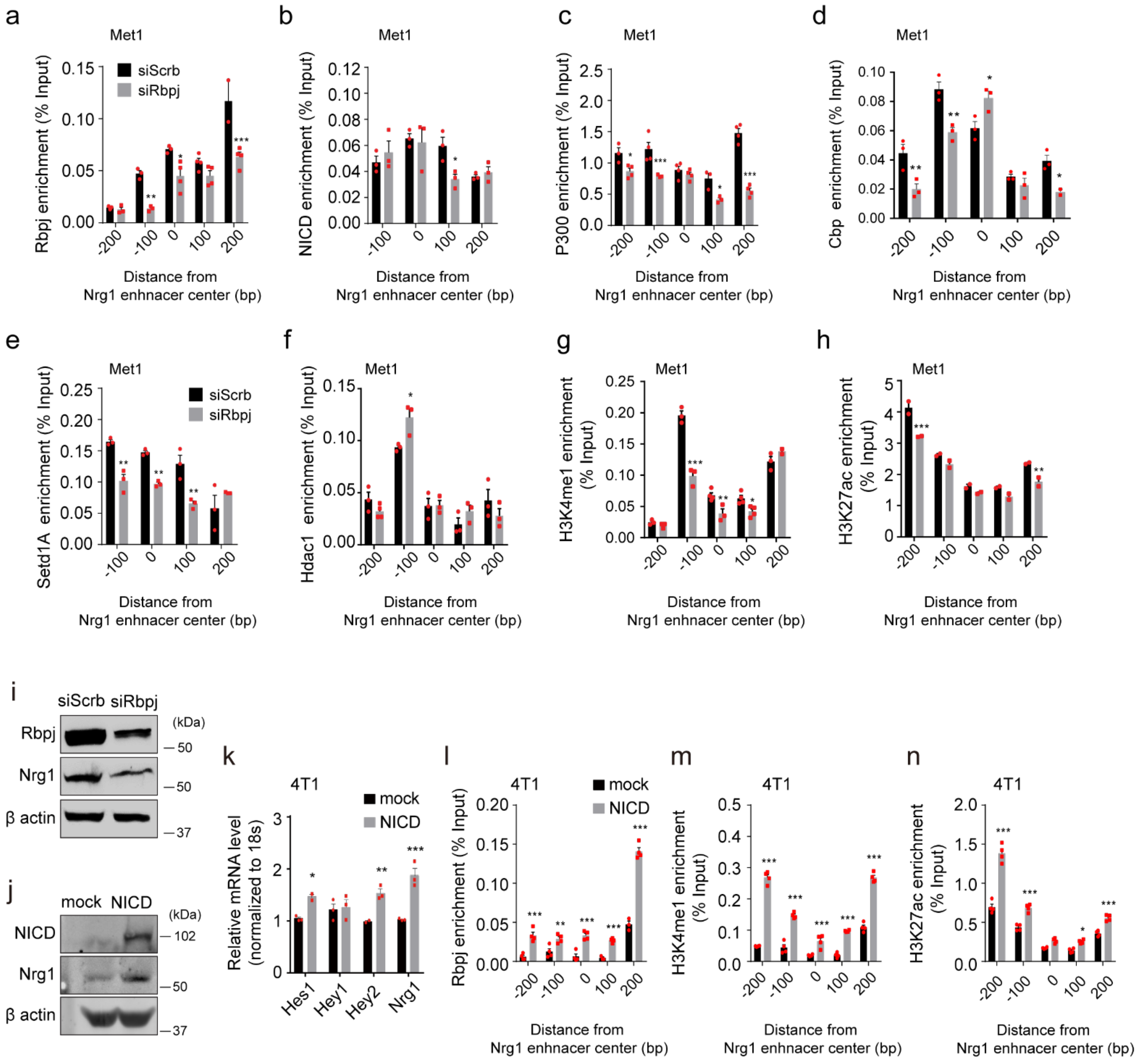

Supplementary Figure 6. Notch dependent assembly of Nrg1 enhanceosome complex. (a-h) ChIP-qPCR analysis of binding occupancy of (a) Rbpj, (b) NICD, (c) P300, (d) CBP, (e) SETD1A, (f) HDAC1 and (g-h) binding enrichment of H3K4me1 and H3K27ac within the Nrg1 enhancer in HG-treated Met1 cells transfected with either scrambled or Rbpj-targeting siRNA. While HDAC1 dissociation was prevented by RBPJ silencing, binding occupancy of NICD, P300, CBP, and SETD1A was decreased with in the enhancer region, accompanied with diminished enrichment of H3K4me1, and H3K27ac. (i) Western blot analysis of Rbpj and Nrg1 protein levels in Met1 cells. (j-k) 4T1 breast cancer cells were transfected with either control or NICD under LG conditions, revealing that NICD and Nrg1 protein levels and mRNA levels

of Nrg1 and Notch target genes were increased by NICD overexpression. (l-n) Binding enrichment of (l) Rbpj, (m) H3K4me1, and (n) H3K27ac within the Nrg1 enhancer were elevated by NICD overexpression in 4T1 cells, as determined by ChIP-qPCR analysis. Data represented mean ±SEM of technical replicates in **a-h, k-n,** and n = 3 independent experiments were performed. Statistical significance was evaluated using two-way ANOVA followed by Bonferroni's **a-h, k-n** post hoc test. *P < 0.05; **P < 0.01; ***P < 0.001, **a** (**P=0.0016, *P=0.0112, ***P<0.0001), **b** (*P=0.0433), **c** (from left to right, *P=0.0281, ***P=0.0007, *P=0.0167, ***P<0.0001), **d** (from left to right, **P=0.0031, ***P=0.0005, *P=0.0139, *P=0.0252), **e** (from left to right, **P=0.0015, **P=0.0093, **P=0.013), **f** (*P=0.0275), **g** (***P<0.0001, **P=0.0053, *P=0.0377), **h** (***P=0.0002, **P=0.0063), **k** (*P=0.0489, **P=0.0082, ***P<0.0001), **l** (from left to right, ***P<0.0001, **P=0.0059, ***P<0.0001, ***P=0.0004, ***P<0.0001), **m** (all ***P<0.0001), **n** (from left to right, ***P<0.0001, ***P<0.0001, *P=0.0261, ***P<0.0001). Source data are provided in Source Data file.

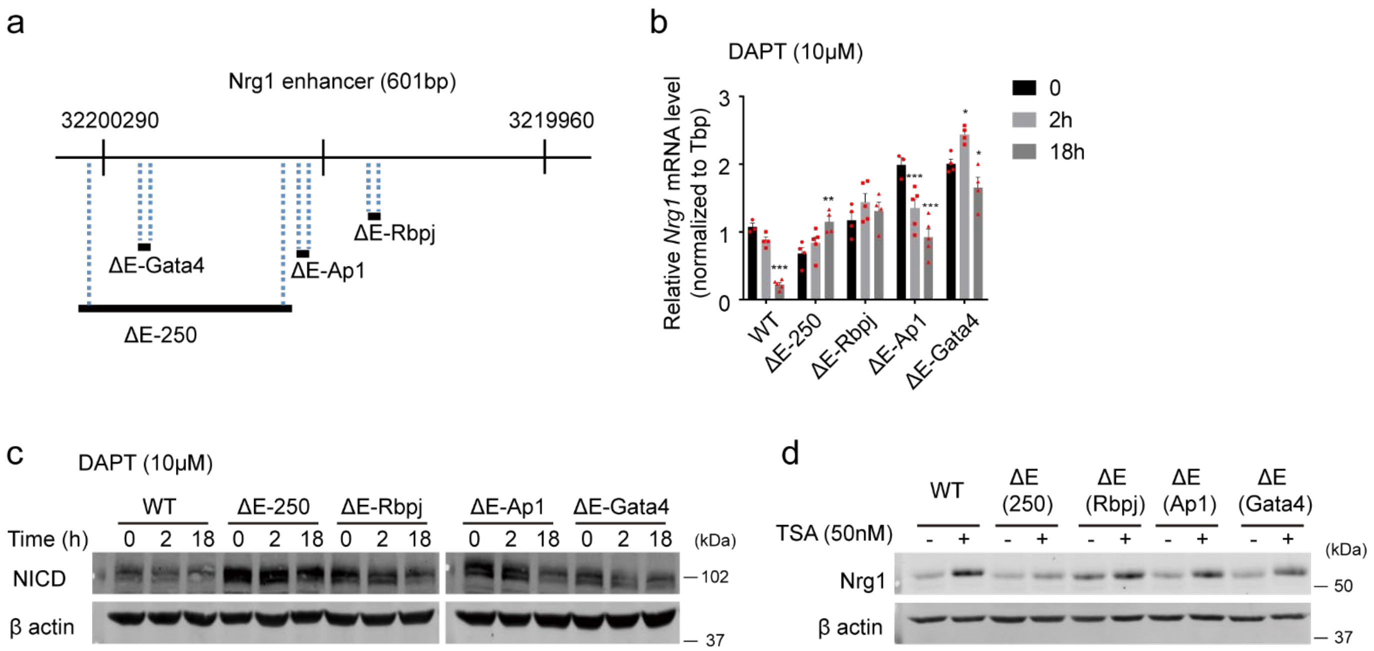

Supplementary Figure 7. Nrg1 Enhancer elements as dictators for HG-mediated signals and Nrg1 overexpression. (a) *Nrg1* enhancer region edited by CRISPR/Cas9 was graphically represented. **(b-c)** Enhancer edited Eo771 cancer cells were treated with DAPT for 18hour. (b) DAPT treatment led to reduction in mRNA level of *Nrg1* in WT cells, but ΔE-250bp and ΔE-Rbpj cells did not display decreased *Nrg1* levels by DAPT treatment. (c) Inhibitory effects of DAPT were confirmed by diminished level of NICD as determined by western blot analysis. **(d)** Either TSA or vehicle were treated to the enhancer edited Eo771 cancer cells from LG. Protein level of NRG1 was found to be upregulated by TSA in WT cell, but those effect was compromised in ΔE-250bp and ΔE-Rbpj cells, suggesting that deletion of the Nrg1 enhancer elements was sufficient to collapse the integration of HG-induced Notch signal at the *Nrg1* enhancer. Data represented mean ±SEM of technical replicates in **b,** and n = 3 independent experiments were performed. Statistical significances were evaluated by *two-way ANOVA* followed by Dunnett's **b** post hoc test. *p < 0.05; **p < 0.01; ***p < 0.001, **b** (from left to right, ***P<0.0001, **P=0.0060, ***P=0.0003, ***P<0.0001, *P=0.0113, *P=0.0426). Source data are provided in Source Data file.

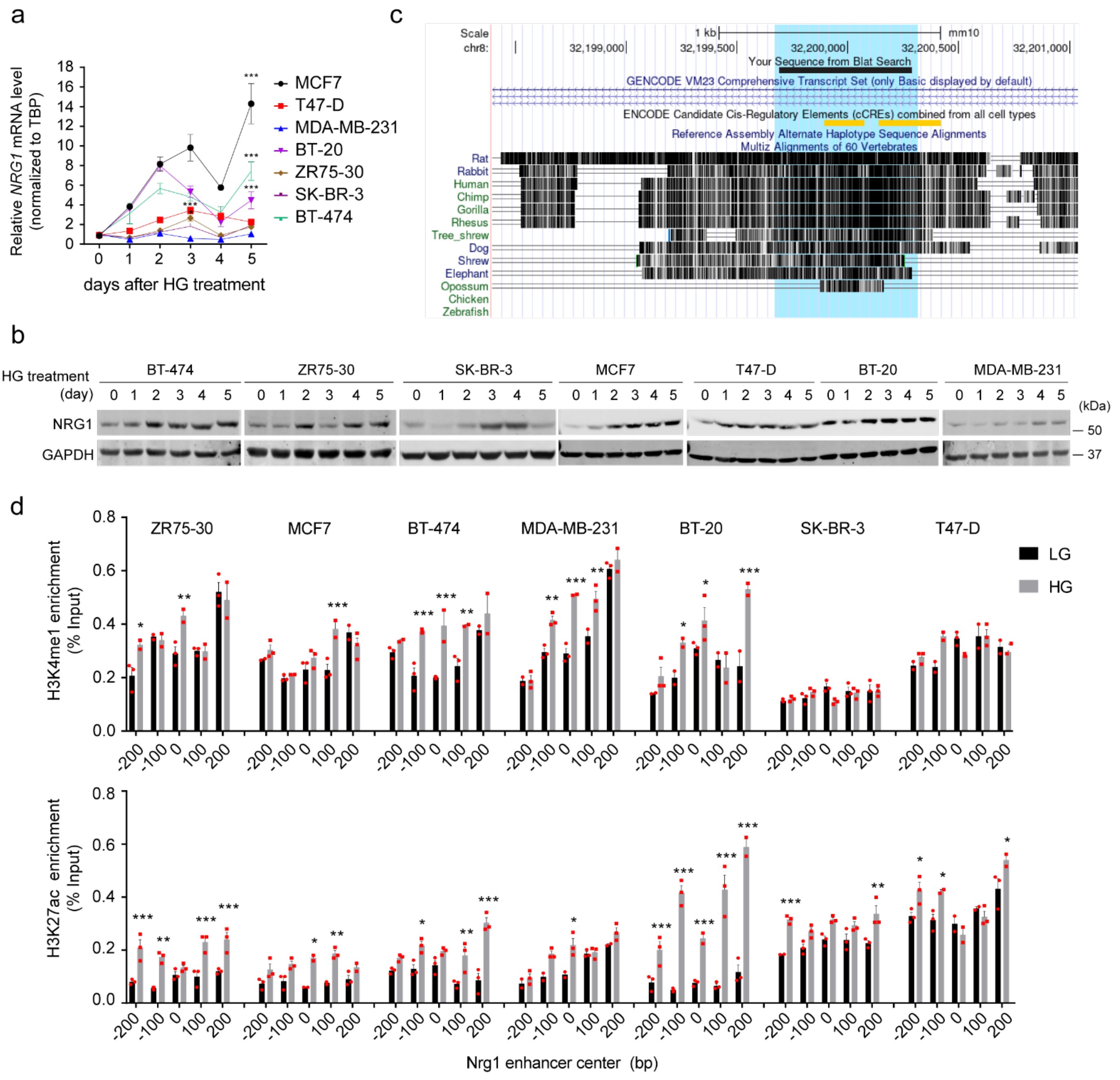

Supplementary Figure 8. HG-induced *NRG1* enhancer activation and NRG1 overexpression in human breast cancer cells. (a-b) Human breast cancer cells were treated with HG for 5 days. Both mRNA and protein levels of NRG1 were found to be upregulated by HG in most cell, except the BT-20 and MDA-MB-231 cells, indicating that HG-induced NRG1 overexpression is well recapitulated in human cancer cell models. **(c)** Alignment of *NRG1* enhancer region among the various species. The *NRG1* enhancer regions were highlighted as light blue, showing that the enhancer is well conserved. **(d)** Human breast cancer cells were treated with either LG or HG for 3 days, and enrichment of active histone marks (H3K4me1 and

H3K27ac) within the *NRG1* enhancer region were determined by ChIP-qPCR. Compared to H3K4me1, H3K27ac was found to be increased by HG treatment in overall enhancer regions, which contributes NRG1 overexpression. Primers used in ChIP-qPCR analysis were listed in **Supplementary Table 4.** Data represented mean ±SEM of technical replicates in **a, d,** and n = 3 independent experiments were performed. Statistical significances were evaluated by *one-way ANOVA* followed by Dunnett's **a** post hoc test, and *two-way ANOVA* followed by Bonferroni's **d** post hoc test. *p < 0.05; **p < 0.01; ***p < 0.001, **a** (from top to bottom, ***P<0.0001, ***P=0.0010, ***P<0.0001, *P=0.0314, ***P<0.0001), and **d** (from left to right, for H3K4me1 *P=0.0452, **P=0.0036, ***P=0.0010, ***P<0.0001, ***P<0.0001, **P=0.0012, **P=0.0072, ***P<0.0001, **P=0.0045, *P=0.0324, *P=0.0441, ***P<0.0001, and for H3k27ac ***P<0.0001, **P=0.0010, ***P<0.0001, ***P=0.0006, *P=0.0391, **P=0.0027, *P=0.0450, **P=0.0040, ***P<0.0001, *P=0.0108, ***P=0.0005, ***P<0.0001, ***P<0.0001, ***P<0.0001, ***P<0.0001, ***P=0.0009, **P=0.0026, *P=0.0133, *P=0.0157, *P=0.0173). Source data are provided in Source Data file.

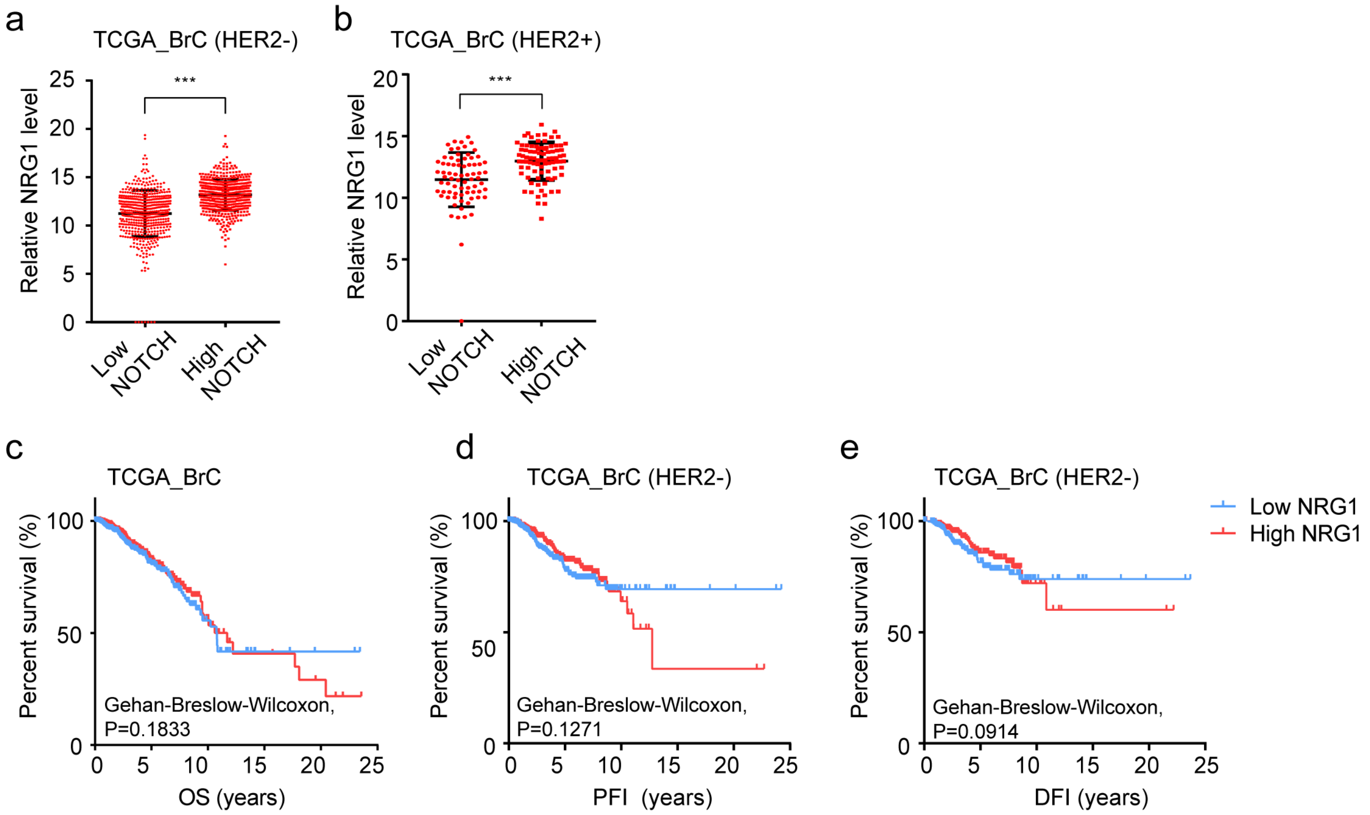

Supplementary Figure 9. Prognostic value of *NRG1* in HER2-positive breast cancer patients. (a-b) NOTCH activity score was given to whole TCGA cohorts and the patients who had above median score were tagged as high-NOTCH group and others as low-NOTCH group. HER2-negative or -positive patients from TCGA breast cancer cohort were subdivided by initial NOTCH label. (a) In both HER2- negative patients (n=463 for low-NOTCH, and n=453 for high-NOTCH) and (b) HER2-positive patients (n=74 for low-NOTCH, and n=87 for high-NOTCH), *NRG1* levels were found to be elevated in high-NOTCH group, regardless of HER2 status. **(c)** Similarly, patients who had above median level of *NRG1* were tagged as high-*NRG1* group and others as low-*NRG1* group. Kaplan-Meier survival analysis revealed that there is no significant difference in overall survival of the low-*NRG1* (n=538) or high-*NRG1* (n=539) groups from the TCGA breast cancer cohort. **(d-e)** HER2-negative patients from TCGA breast cancer cohort were subdivided by the NRG1 label. Kaplan-Meier survival analysis revealed that there are no significant differences in PFI and DFI of the low-*NRG1* (n=467) or high-*NRG1* (n=449) patient groups from the TCGA HER2-negative breast cancer cohort, suggesting that NRG1 could not significantly predict clinical outcome in HER2-negative patients. Data represented mean ±SEM of technical replicates in **a, b,** and results shown in **a-e** are derived from single analysis (n = 1). Statistical significances were evaluated by two-tailed Student's t-test **a**, **b**, and two-tailed Gehan-Breslow-Wilcoxon test **c-e**. $^*p < 0.05$; $^{**}p < 0.01$; $^{***}p < 0.001$,

a (***P<0.0001), **b** (***P<0.0001), **c-e** (c to e, P=0.1833, P=0.1271, P=0.0914). Source data are provided in Source Data file.

Supplementary table 1. siRNA sequences used for transfection

siRNA targets	Sequence (5' to 3')
Nfia	GCACAUAACUCAAAUGAAA
Gata4	GGACAUAAUCACCGCGUAA
Rbpj	GCACAGAAGUCUUACGGAA
Prrx1	AGGAGAAGAAGAAGAGAAA
Ctbp1	GAGAAGAUCUGGAGAAGUU
P300	GGCUUGACUUCUCCAAACA
Cbp	CCUAUCCGAGCAAACAUCA
Setd1A	CGAAUGAAGUACUAUGAAU

Supplementary table 2. shRNA sequences used in target gene knockdown

shRNA targets	Sequence (5' to 3')	direction
Scrambled	CCTAAGGTTAAGTCGCCCTCG	Sense
	CGAGGGCGACTTAACCTTAGG	Antisense
P300	CCCTGGATTAAGTTTGATAAA	Sense
	TTTATCAAACTTAATCCAGGG	Antisense
Cbp	CGCGAATGACAACACAGATTT	Sense
	AAATCTGTGTTGTCATTCGCG	Antisense
Setd1A	CGGCGGTTACTAAGCGCTATT	Sense
	AATAGCGCTTAGTAACCGCCG	Antisense
Nrg1	CCCAGATTGAAAGAGATGAAA	Sense
	TTTCATCTCTTTCAATCTGGG	Antisense

Supplementary table 3. Primer sequences used for RT-qPCR

qPCR primer	Sequence (5' to 3')	direction
18s	AGGGTTCGATTCCGGAGAGG	Forward
	CAACTTTAATATACGCTATTGG	Reverse
Ccnd1	AGCCTCCAGAGGGCTGTCGG	Forward
	GGCTGTGGTCTCGGTTGGGC	Reverse
Ctbp1	GTGCCCTGATGTACCATACCA	Forward
	TGATGTCGATATTGTCAAACCCG	Reverse
Dll1	GACCTCGCAACAGAAAACCCA	Forward
	TTCTCCGTAGTAGTGCTCGTC	Reverse
Dll3	CTGGTGTCTTCGAGCTACAAAT	Forward
	TGCTCCGTATAGACCGGGAC	Reverse
Dll4	TTCCAGGCAACCTTCTCCGA	Forward
	ACTGCCGCTATTCTTGTCCC	Reverse
Gata4	CCCTACCCAGCCTACATGG	Forward
	ACATATCGAGATTGGGGTGTCT	Reverse
Hes1	TCAACACGACACCGGACAAA	Forward
	CTTGGAATGCCGGGAGCTAT	Reverse
Hes5	AGTCCCAAGGAGAAAAACCGA	Forward
	GCTGTGTTTCAGGTAGCTGAC	Reverse
Hey1	GCCTGGTCTCCCATCTCAAC	Forward
	TGTGTGGGTGATGTCCGAAG	Reverse
Hey2	TCCAGGCTACAGGGGGTAAA	Forward
	AGATGAGAGACAAGGCGCAC	Reverse
Jag1	CCTCGGGTCAGTTTGAGCTG	Forward
	CCTTGAGGCACACTTTGAAGTA	Reverse
Jag2	CTGTGCAGCGTGTTCAGTG	Forward
	GTGTCCACCATACGCAGATAAC	Reverse
Nfia	CTGACAGCGTAATGGCAGGG	Forward
	GGCCGAGATGTCGTAGCAAA	Reverse
Nrg1	GGGAATGAGCTGAACCGTAG	Forward
	ACAATGGTGATGTTGGCAGA	Reverse
NRG1	CTACATCTACATCCACCACTGG	Forward
	TTGCACAAGTATCTCGAGGGGT	Reverse
Prrx	GAGCGTGTCTTTGAGCGGA	Forward
	CATGTGGCAGAATAAGTAGCCAT	Reverse
Rbpj	CTCCACCCAAACGACTCACTA	Forward
	TCCAACCACTGCCCATAAGATA	Reverse
Tbp	CTTCCTGCCACAATGTCACAG	Forward
	CCTTTCTCATGCTTGCTTCTCTG	Reverse
TBP	CCACTCACAGACTCTCACAAC	Forward
	CTGCGGTACAATCCCAGAACT	Reverse

Supplementary table 4. Primer sequences used for ChIP assay

ChIP Primer	Sequence (5' to 3')	direction
Mouse Nrg1 enhancer (+200)	AACTTATCTATATTTCCCCTCTCTTTACTCAAAAC	Forward
	ATGAGAAATGGGAAGGAAGTTGAAGA	Reverse
Mouse Nrg1 enhancer (+100)	AAACATTAATAATCAAATTTCACAATTACTTTGTTGGC	Forward
	TTCCATAGCCCTTAGGCATGTTAGA	Reverse
Mouse Nrg1 enhancer (0)	GCCTTCGTTTTTTTATGAAGTGGGG	Forward
	TGCAAGGCCTGCCACT	Reverse
Mouse Nrg1 enhancer (-100)	TGCCACAGCTTTTGTTTTTCTTAGC	Forward
	AGGGCACTTGCACGGAA	Reverse
Mouse Nrg1 enhancer (-200)	GAATCTTTCTTTTGTCAATGTCTGGATAATCAG	Forward
	AATGTGTTGGATTGGAATTGTGAAAGC	Reverse
Human Nrg1 enhancer (+200)	CCT GCT ACG GAG ATT TGC CA	Forward
	TCA AAT GGG AAG GAA GTA GAA TAG C	Reverse
Human Nrg1 enhancer (+100)	GTT TGT GGT AGA GAA CAG AAA ACA G	Forward
	GCA TGT AAG ATC TTG GTA CTT TCC C	Reverse
Human Nrg1 enhancer (0)	CCT TCC CAT TTG ACA TAT CTT GCT	Forward
	AGG CTC TCC AAG GTT TGC CA	Reverse
Human Nrg1 enhancer (-100)	AAA GGC TCT GGA ATG TCG TG	Forward
	CAC TTT CCA GAG GCC ACT TC	Reverse
Human Nrg1 enhancer (-200)	ATG GCA AAC CTT GGA GAG CC	Forward
	TGG ATT GGA ATC ATG AAA ACT GTC T	Reverse

Supplementary table 5. Primer sequences used for cloning Nrg1 enhancer and promoter

Enhancer/promoter	Sequence (5' to 3')	direction
+100 bp	AAACATTAATAATCAAATTTCACAATTACTTTGTTGGC	Forward
	TTCCATAGCCCTTAGGCATGTTAGA	Reverse
+200 bp	AACTTATCTATATTTCCCCTCTCTTTACTCAAAAC	Forward
	ATGAGAAATGGGAAGGAAGTTGAAGA	Reverse
-1316 bp	CAG CCA CGG GTC TGT TAG TA	Forward
	GCC AAC TTA GCC TGC TCT TT	Reverse
-116 bp	GTG GTG GGG AAA GAG GGA G	Forward
	TCA CAG TCC GGT CTA TGC TC	Reverse

Supplementary table 6. Oligomer sequences used for guide RNA

sgRNA Primer	Sequence (5' to 3')	direction
Non-target	caccgGCGAGGTATTCGGCTCCGCG	Forward
	aaacCGCGGAGCCGAATACCTCGCc	Reverse
Rbpj	caccgCGAAGGCATGAGAAATGGGA	Forward
	aaacTCCCATTTCTCATGCCTTCGc	Reverse
250bp (Nrg1 enhancer)	caccgAAGGCAGCAGAAGAATGTGT	Forward
	aaacCTTATCGCGAATATGGAAAGc	Reverse
	caccgTCAACAATCAGGGTCAGAGT	Forward

	aaacACTCTGACCCTGATTGTTGAc	Reverse
Ap1	caccgGTCCTGGCTCAGATGATTCA	Forward
	aaacTGAATCATCTGAGCCAGGACc	Reverse
Gata4	caccgCTTTCCATATTCGCGATAAG	Forward
	aaacCTTATCGCGAATATGGAAAGc	Reverse

Supplementary table 7. Primer sequences used for bisulfite sequencing, enhancer bait for pull-down assay

**Methylation Primer**	**Sequence (5' to 3')**	**direction**
mNrg1 enhancer (-100) Methylation	TTTTTATTTTGTTTGTTTTTGTTGG	Forward
	AAACTTTACCAATTCTTTCTCCTTAAC	Reverse
mNrg1 enhancer (+200) Methylation	TTAGGGGTTTTGGAGGATATTTATT	Forward
	TTAAAATTATAAAAACTTTCCACTTATC	Reverse

**Enhancer-bait Primer**	**Sequence (5' to 3')**	**direction**
Oligomer bait	TTTCTTAGCTAGAAGAGATGCAAATATTTAACCA	Forward
	CAAGGCCCCTGATTATCCAGAC	Reverse

Supplementary table 8. Information of HER2-positive breast cancer patients

ID	ER	PR	HER2	Ki-67	PNM stage	chemothrapy	Hormonal therapy	HER2 targeted therapy	Diabetic status (at biopsy)
2	-	-	3+	50	pT2N2M0	AC-T#8(2006.3.2-2006.7.31)	no	Lapatinib (1Y)	euglycemia
3	-	-	3+	5	pT2N0M0	CMF#6(2006.5.4-2006.10.12)	no	no	euglycemia
4	-	-	3+	20	pT2N0M0	CMF#6(2006.5.4 -2006.10.12)	no	no	hyperglycemia
5	+	+	3+	1	pTisN0M0	no	tamoxifen →fareston	no	euglycemia
6	-	-	3+	15	pT2N0M0	FEC#6(2006.5.17-2006.8.30)	no	no	hyperglycemia
7	+	+	3+	20	pT2N0M0	FEC#6(2006.5.29-2006.10.9)	arimidex	lapatinib	euglycemia
9	-	+	3+	1	pT1N0M0	FAC#6(2007.4.8-2007.8.13)	tamoxifen	no	euglycemia
10	-	-	2+	3	pT2N0M0	CMF#6(2008.1.28-7.3)	no	no	euglycemia
11	-	+	2+	3	pT1N1M0	AC-T#8(2008.3.19-2008.6.11)	tamoxifen	herceptin → lapatinib	euglycemia
12	-	-	3+	10	pT2N1M0	AC-T#8(2008.4.2-9.29)	no	herceptin	hyperglycemia
13	-	-	3+	10	pT2N1M0	AC-D#8(2008.4.8-9.18)	no	no	euglycemia
14	+	+	2+	15	pT2N0M0	FAC#6(2008.4.16-8.26)	tamoxifen->arimidex	no	euglycemia
15	-	-	2+	5	pT2N0M0	AC#4(2008.5.20-7.31)	no	no	hyperglycemia
16	-	-	3+	5	pT1N1M0	AC-D#8(2008.5.27-10.28)	no	herceptin	hyperglycemia
17	-	+	2+	2	pT2N2M0	AC-T#8(2008.6.18-11.19)	no	herceptin+lapatinib	hyperglycemia
18	-	-	2+	10	pT2N0M0	FAC#6(2008.7.14-12.10)	no	herceptin	euglycemia
19	-	-	3+	5	pT2N0M0	FAC#6(2008.7.24-11.20)	no	herceptin	euglycemia
20	-	-	2+	25	pT2N0M0	FAC#6(2008.7.24-11.27)	no	herceptin	euglycemia
21	-	-	3+	7	pT1N0M0	FAC#6(2008.9.4-2009.1.20)	no	no	euglycemia
22	-	-	3+	8	pT2N1M0	AC-D#8(2008.8.19-2009.1.29)	no	no	hyperglycemia
23	+	+	3+	5	pT1N0M0	-	letrozole	no	hyperglycemia
24	-	-	2+	3	pT1N1M0	AC-D#8	no	herceptin#4	euglycemia
25	+	+	2+	7	pT1N1M0	AC-T#8(2008.10.13-2009.3.30)	tamoxifen	Herceptin (1Y)	euglycemia
26	+	+	2+	5	cT3N2M1	palliative GN#10(2008.12.10-2009.10.14) →CMF#1(2009.12.21)		lapatinib+xeloda#4	hyperglycemia
28	-	-	3+	15	pT2N1M0	AC-D#8(2009.1.20-6.20)	no	no	euglycemia
29	+	-	2+	15	pT3N3M0	AC-T#8(2009.1.7-7.15)	tamoxifen	herceptin	euglycemia
30	+	+	3+	20	pT4N3M0	AC-T#8(2009.1.21-7.27)	tamoxifen	no	euglycemia
