# Supplementary material for: Epigenetic regulation of Neuregulin 1 promotes breast cancer progression associated to hyperglycemia
Source: Nat Commun. 2023 Jan 27;14:439. doi: 10.1038/s41467-023-36179-8 (PMC9883495; doi:10.1038/s41467-023-36179-8)
Supplement: Supplementary file 2 — Reporting Summary [file 41467_2023_36179_MOESM2_ESM.pdf]

nature portfolio

Corresponding author(s): Jiyoung Park
Last updated by author(s): Dec 12, 2022

Reporting Summary

Nature Portfolio wishes to improve the reproducibility of the work that we publish. This form provides structure for consistency and transparency in reporting. For further information on Nature Portfolio policies, see our Editorial Policies and the Editorial Policy Checklist.

Statistics

For all statistical analyses, confirm that the following items are present in the figure legend, table legend, main text, or Methods section.

n/a	Confirmed	
☐	☒	The exact sample size (*n*) for each experimental group/condition, given as a discrete number and unit of measurement
☐	☒	A statement on whether measurements were taken from distinct samples or whether the same sample was measured repeatedly
☐	☒	The statistical test(s) used AND whether they are one- or two-sided<br>*Only common tests should be described solely by name; describe more complex techniques in the Methods section.*
☒	☐	A description of all covariates tested
☒	☐	A description of any assumptions or corrections, such as tests of normality and adjustment for multiple comparisons
☐	☒	A full description of the statistical parameters including central tendency (e.g. means) or other basic estimates (e.g. regression coefficient) AND variation (e.g. standard deviation) or associated estimates of uncertainty (e.g. confidence intervals)
☒	☐	For null hypothesis testing, the test statistic (e.g. *F*, *t*, *r*) with confidence intervals, effect sizes, degrees of freedom and *P* value noted<br>*Give P values as exact values whenever suitable.*
☐	☒	For Bayesian analysis, information on the choice of priors and Markov chain Monte Carlo settings
☒	☐	For hierarchical and complex designs, identification of the appropriate level for tests and full reporting of outcomes
☐	☒	Estimates of effect sizes (e.g. Cohen's *d*, Pearson's *r*), indicating how they were calculated

Our web collection on statistics for biologists contains articles on many of the points above.

Software and code

Policy information about availability of computer code

Data collection: MS/MS samples were analyzed on the Sequest Sorcerer platform (Sagen-N Research, San Jose, CA). Sequest was set up to interrogate the Mus musculus protein database (17278 entries, UniProt (http://www.uniprot. org/))

Survival annotations and gene expression data of breast cancer patients were downloaded from TCGA database.

Data analysis	No custom codes were developed in this study.<br><br>Scaffold v5.0.1 was used to validate MS/MS-based peptide and protein identities. Peptide identities were accepted if they had > 94.0% probability of achieving <1.0% FDR based on the Peptide Prophet algorithm with Scaffold delta-mass correction. Protein identities were accepted if they had > 99.0% probability of achieving < 1.0% FDR and contained at least two identified peptides. Protein probabilities were assigned using the Protein Prophet algorithm v5.0<br><br>R software v4.0.2 was used to analyze TCGA breast cancer patients. Singscore_1.8.0 (R package) was used to assign the NOTCH activity. After arranging data, statistical analyses were conducted using GraphPad Prism 7.<br><br>Incucyte software v2011A was used to collect images and confluency data of the cancer cells.<br>Li-cor Imagestudio software v5.2 was used to collect western blot images.<br>QunatStudio (appliedbiosystem) v1.5.1 was used to collect raw qPCR results.

For manuscripts utilizing custom algorithms or software that are central to the research but not yet described in published literature, software must be made available to editors and reviewers. We strongly encourage code deposition in a community repository (e.g. GitHub). See the Nature Portfolio guidelines for submitting code & software for further information.

Data

Policy information about availability of data

All manuscripts must include a data availability statement. This statement should provide the following information, where applicable:

- Accession codes, unique identifiers, or web links for publicly available datasets
- A description of any restrictions on data availability
- For clinical datasets or third party data, please ensure that the statement adheres to our policy

Survival annotations and gene expression data of breast cancer patients could be obtained from TCGA database (TCGA-BRCA project, dbGaPaccessionphs000178)
The datasets are freely available and accessible at https://portal.gdc.cancer.gov/projects/TCGA-BRCA
Survival annotations also could be accessible at https://doi.org/10.1016/j.cell.2018.02.052

Mus musculus protein database used for Protein Propher algorithm was obtained from Uniprot database (GCA_000001635.8 from Ensembl).
The datasets are freely accessible at https://www.uniprot.org/proteomes/UP000000589

Data sets generated from current study are available from the corresponding author.
Proteomics data of Nrg1 enhancer binding proteins is accessible at https://www.ebi.ac.uk/pride/ (accession number; 000000000000000)

Human research participants

Policy information about studies involving human research participants and Sex and Gender in Research.

Reporting on sex and gender	All patients were female, and their gender was not considered in this study.
Population characteristics	Korean female breast cancer patients (over age 20) who had breast cancer treatment<br><br>There are confounding factors including status of chemotherapy, Hormonal therapy, HER2 targeted therapy
Recruitment	HER2-positive breast cancer patients, enrolled in Seoul National University Hospital<br>There is no potential of biases for collecting HER2-positive breast cancer cohort.
Ethics oversight	Seoul National University Hospital committee (2111-069-1271)

Note that full information on the approval of the study protocol must also be provided in the manuscript.

Field-specific reporting

Please select the one below that is the best fit for your research. If you are not sure, read the appropriate sections before making your selection.

☒ Life sciences ☐ Behavioural & social sciences ☐ Ecological, evolutionary & environmental sciences

For a reference copy of the document with all sections, see nature.com/documents/nr-reporting-summary-flat.pdf

Life sciences study design

All studies must disclose on these points even when the disclosure is negative.

Sample size	For statistical test, 3 technical replicates were tested in most of samples from cell culture experiments, while some sets were tested as duplicate. In tumor allograft mice models, at least 5 samples were allocated in each group. Calculation of sample size was not performed. Sample sizes were determined based on similar experiments from previous reports.
Data exclusions	Data exclusions were applied when the samples used in qPCR analysis had high standard deviation (over 0.3) in replicate group.<br>During the drug treatments experiment, tumor allograft samples were excluded when tumors failed to develop or when tumors development is too fast (over 1 g after dissection).<br>No data was excluded in samples from human breast cancer patients.<br>Exclusion criteria were not pre-established by others.
Replication	At least 3 sets of experiments were carried out to confirm the reproducibility of the results, and we got consistent results.
Randomization	All the experimental groups were randomly allocated in in vitro experiments, before treatment.<br>Breast cancer patients specimens were divided into euglycemic or hyperglycemic group, depending on their blood glucose level.<br>Mice were randomly divided into 3 groups and injected with 3 different CRISPR-edited cells.<br>Mice were randomly divided into 2 groups and injected with either vehicle or Streptozotocin (STZ).<br>Either vehicle or STZ treated mice were randomly divided into 4 groups and they were treated with following arms; vehicle, Lapatinib, DAPT, Lapatinib + DAPT
Blinding	Data collection and analysis were under complete blindness in the double immunofluorescence (Notch, O-GlcNAc) of breast cancer cells.<br>Data collection and analysis were under complete blindness in the NICD immunofluorescence of human breast cancer patients<br>In other case, blindness was not necessary for group allocation during data collection and/or analysis, as the data was processed in semi-autmomatic manner. (There was no room for self-biases during the process)

Reporting for specific materials, systems and methods

We require information from authors about some types of materials, experimental systems and methods used in many studies. Here, indicate whether each material, system or method listed is relevant to your study. If you are not sure if a list item applies to your research, read the appropriate section before selecting a response.

Materials & experimental systems

n/a	Involved in the study	
☐	☒	Antibodies
☐	☒	Eukaryotic cell lines
☒	☐	Palaeontology and archaeology
☐	☒	Animals and other organisms
☒	☐	Clinical data
☒	☐	Dual use research of concern

Methods

n/a	Involved in the study	
☒	☐	ChIP-seq
☒	☐	Flow cytometry
☒	☐	MRI-based neuroimaging

Antibodies

Antibodies used

H3K4me1 (Abcam, ab8895 - GR291358-1, 2ug for ChIP reaction),
H3K27ac (Abcam, ab4729 - GR261979-1, 2ug for ChIP reaction),
NRG1 (Abcam, ab27303 - , 1:800 dilution in Western blot(WB)),
NOTCH1 (Abcam, ab27526 - GR3229344-3, 1:1,000 dilution in WB, 1:100 in ChIP, 1:100 in immunofluorescence),
RBPJ (Abcam, ab25949 - GR291358-1, 1:1,000 dilution in WB, 2μg in ChIP),
O-GlcNAc (Abcam, ab2735 - GR267084-8, 1:1,000 dilution in WB, 1:200 in immunofluorescence),
P300 (Abcam, ab275378 [EPR23495-268] - GR3359600-9, 1:1,000 dilution in WB, 1:100 in ChIP),
CBP (CST, #7389 [D6C5] - 06/2020 Lot1, 1:1,000 dilution in WB, 1:100 in ChIP),
HDAC1 (CST, #34589 [D5C6U] - 01/2019 Lot1, 1:1,000 dilution in WB, 1:50 in ChIP),
SETD1A (CST, #50805 [E3E2S] - 01/2020 Lot1, 1:1,000 dilution in WB, 1:50 in ChIP),
GAPDH (SCBT, sc-32233 [6C5], 1:1,000 dilution in WB),
Lamin A/C (SCBT, sc-376248 [E-1] A2121, 1:1,000 dilution in WB),
β-Actin (SCBT, sc-47778 [C4] A2315, 1:1,000 dilution in WB).

IR-dye 680 anti-mouse (Li-cor, P/N: 926-68070, Lot No. D01014-04, 1:15,000 dilution)
IR-dye 800 anti-mouse (Li-cor, P/N: 926-32210, Lot No. c91210-09, 1:15,000 dilution)
IR-dye 800 anti-rabbit (Li-cor, P/N: 926-32211, Lot No. D01110-10, 1:15,000 dilution)
Alexa488 anti-rabbit (invitrogen, A21206, Lot 2289872, 1:1,000 dilution)

Alexa594 anti-mouse (invitrogen, A11005, Lot 2043369), 1:2,000 dilution

Validation

H3K4me1 (Abcam, ab8895 - Rabbit polyclonal to Histone H3 (mono methyl K4), our Abpromise guarantee covers the use of ab8895 in the following tested applications; ChIP Use 2 µg for 25 µg of chromatin. We recommend Myo-D ChIP primer pair ab269261 as positive control.

H3K27ac (Abcam, ab4729 - Rabbit polyclonal to Histone H3 (acetyl K27), our Abpromise guarantee covers the use of ab4729 in the following tested applications; ChIP Use 2 µg for 25 µg of chromatin. We recommend GAPDH positive control ChIP primer pair ab267832 as a positive control.

NRG1 (Abcam, ab27303 - Rabbit polyclonal to NRG1 type I, our Abpromise guarantee covers the use of ab27303 in the following tested applications; WB Use a concentration of 1 µg/ml. Detects a band of approximately 51 kDa (predicted molecular weight: 70 kDa).

NOTCH1 (Abcam, ab27526 - Rabbit polyclonal to Notch1, our Abpromise guarantee covers the use of ab27526 in the following tested applications; WB Use at an assay dependent concentration. PubMed: 18156632, ChIP Use at an assay dependent concentration. IHC-P 1/200. for 10 minutes at room temperature. Staining of Formalin-fixed tissues requires boiling tissue sections in 10mM citrate buffer, pH 6.0 for 10 minutes followed by cooling at room temperature for 20 minutes.

RBPJ (Abcam, ab25949, Rabbit polyclonal to RBPJK, our Abpromise guarantee covers the use of ab25949 in the following tested applications; WB Use a concentration of 1 µg/ml. Detects a band of approximately 56 kDa (predicted molecular weight: 56kDa).

O-GlcNAc (Abcam, ab2735 - Mouse monoclonal [HGAC85] to O-Linked N-Acetylglucosamine, our Abpromise guarantee covers the use of ab2735 in the following tested applications; WB Use a concentration of 1 µg/ml. This antibody detects several proteins representing O-GlcNAc glycoproteins

P300 (Abcam, ab275378 - Rabbit monoclonal [EPR23495-268] to KAT3B / p300, our Abpromise guarantee covers the use of ab275378 in the following tested applications; ChIP Use a concentration of 5 µg/ml.

CBP (CST, #7389, D6C5) - CBP (D6C5) Rabbit mAb recognizes endogenous levels of total CBP protein. This antibody does not cross-react with p300 protein. This antibody has been validated using SimpleChIP® Enzymatic Chromatin IP Kits.

HDAC1 (CST, #34589, D5C6U) - HDAC1 (D5C6U) XP® Rabbit mAb recognizes endogenous levels of total HDAC1 protein. This antibody has been validated using SimpleChIP® Enzymatic Chromatin IP Kits.

SETD1A (CST, #50805, E3E2S) - SET1A (E3E2S) Rabbit mAb recognizes endogenous levels of total SET1A protein. This antibody has been validated using SimpleChIP® Enzymatic Chromatin IP Kits

GAPDH (SCBT, sc-32233), GAPDH (6C5) is a mouse monoclonal antibody raised against GAPDH purified from muscle of rabbit origin. The GAPDH (6C5) Antibody was generated using GAPDH, and glyceraldehyde-3-phosphate dehydrogenase as the antigen. It reacts with Human, Mouse, Rat, and Rabbit. This antibody has been shown to work in applications such as: Precipitation, Immunofluorescence, Immunoprecipitation, and Western Blot.

Lamin A/C (SCBT, sc-376248), Lamin A/C (E-1) is a mouse monoclonal antibody specific for an epitope mapping between amino acids 2-29 at the N-terminus of Lamin A/C of human origin. The Lamin A/C (E-1) Antibody was generated using Lamin A/C as the antigen. It reacts with Human, Mouse, and Rat. This antibody has been shown to work in applications such as: EIA, Immunoassay, Precipitation, ELISA, Immunofluorescence, Immunohistochemistry - fixed, Immunoprecipitation, and Western Blot.

β-Actin (SCBT, sc-47778). - β-Actin (C4) is a mouse monoclonal antibody raised against gizzard Actin of chicken origin. The beta-Actin (C4) Antibody has been validated for the following applications: EIA, Immunoassay, Precipitation, ELISA, Immunofluorescence, Immunohistochemistry - fixed, Immunoprecipitation, and Western Blot.
beta-Actin (C4)

Eukaryotic cell lines

Policy information about cell lines and Sex and Gender in Research

Cell line source(s)

Met1 was isolated from MMTV-PyMT mice
Eo771 (CRL-3461) and 4T1 (CRL-2539) breast cancer cell lines were acquired from the ATCC
Human breast cancer cell lines ZR75-30 (CRL-1504), BT-474 (HTB-20), SK-BR-3 (HTB-30), MCF7 (HTB-22), MDA-MB-231 (CRM-HTB-26), BT-20 (HTB-19), and T47-D (HTB-133) were also acquired from ATCC

Authentication

Cell lines were not authenticated.

Mycoplasma contamination

All cell lines were tested negatively for mycoplasma contamination.

Commonly misidentified lines
(See ICLAC register)

BT-20 cell line is under the list of known misidentified cell lines provided from the International Cell Line Authentication Committee (ICLAC). However, BT-20 cell was used as one of the triple negative breast cancer cells, which displayed limited responses to high glucose, and thus, it does not compromise out results.

Animals and other research organisms

Policy information about studies involving animals; ARRIVE guidelines recommended for reporting animal research, and Sex and Gender in Research

Laboratory animals	Wild-type C57BL/6J mice (8week) were purchased from Hyochang Science. Mice were housed in air-filtered flow cabinets with a 12-hours light cycle at 22±2°C and 55±5% humidity, and allowed free access to food and water
Wild animals	This study did not involve wild animals.
Reporting on sex	C57BL/6J mice (8week, wild-type male) were used in allograft model, as the cell-autonomous effect was not related with sex.<br>C57BL/6J mice (8week, wild-type female) were used in drug treatment model, as the breast cancer are rarely diagnosed in male and the therapeutic recipients are most likely to be females.
Field-collected samples	This study did not involve samples collected from the filed.
Ethics oversight	Institutional Animal Care and Use Committee of the Ulsan National Institute of Science and Technology (UNISTIACUC-20-07)

Note that full information on the approval of the study protocol must also be provided in the manuscript.
