# Supplementary material for: Epigenetic regulation of Neuregulin 1 promotes breast cancer progression associated to hyperglycemia
Source: Nat Commun. 2023 Jan 27;14:439. doi: 10.1038/s41467-023-36179-8 (PMC9883495; doi:10.1038/s41467-023-36179-8)
Supplement: Supplementary file 3 — Description of Additional Supplementary Files [file 41467_2023_36179_MOESM3_ESM.docx]

Description of Additional Supplementary Information File

Supplementary Data 1

List of Nrg1 enhancer binding proteins enriched by high glucose treatment, identified by LC-MS/MS analysis.
